# Biophysical characterization of synthetic adhesins for predicting and tuning engineered living material properties

**DOI:** 10.1016/j.matt.2024.03.019

**Published:** 2024-04-22

**Authors:** Stefana A. Costan, Paul M. Ryan, Honesty Kim, Charles W. Wolgemuth, Ingmar H. Riedel-Kruse

**Affiliations:** 1 Department of Molecular and Cellular Biology, University of Arizona, Tucson, AZ 85721, USA; 2 Department of Biomedical Engineering, University of Arizona, Tucson, AZ 85721, USA; 3 Department of Physics, University of Arizona, Tucson, AZ 85721, USA; 4 Department of Applied Mathematics, University of Arizona, Tucson, AZ 85721, USA; 5 Lead contact

## Abstract

Bacterial synthetic multicellular systems are promising platforms for engineered living materials (ELMs) for medical, biosynthesis, environmental, and smart materials applications. Recent advancements in genetically encoded adhesion toolkits have enabled precise manipulation of cell-cell adhesion and the design and patterning of self-assembled multicellular materials. However, in contrast to gene regulation in synthetic biology, the characterization and control of synthetic adhesins remains limited. Here, we demonstrate the quantitative characterization of a bacterial synthetic adhesion toolbox through various biophysical methods. We determine key parameters, including number of adhesins per cell, in-membrane diffusion constant, production and decay rates, and bond-breaking force between adhesins. With these parameters, we demonstrate the bottom-up prediction and quantitative tuning of macroscopic ELM properties (tensile strength) and, furthermore, that cells inside ELMs are connected only by a small fraction of available adhesins. These results enable the rational engineering, characterization, and modeling of other synthetic and natural adhesins and multicellular consortia.

## Graphical Abstract

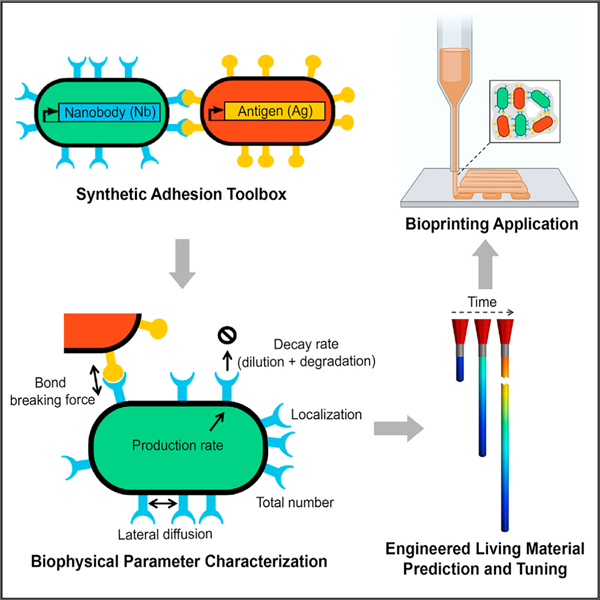

To expand the integration of synthetic biology in material science, we quantitatively characterized a bacterial synthetic adhesion toolbox through several biophysical methods. Specifically, we measured the synthetic adhesins’ turnover rates, their surface density and spatial distribution, their membrane diffusion rate, and their intermolecular bond-breaking force. With these parameters, we then demonstrated how to predict and quantitatively tune the macroscopic properties of engineered living materials consisting of cells expressing these synthetic adhesins, which is essential for applications such as bioprinting.

## INTRODUCTION

**Figure 1.**
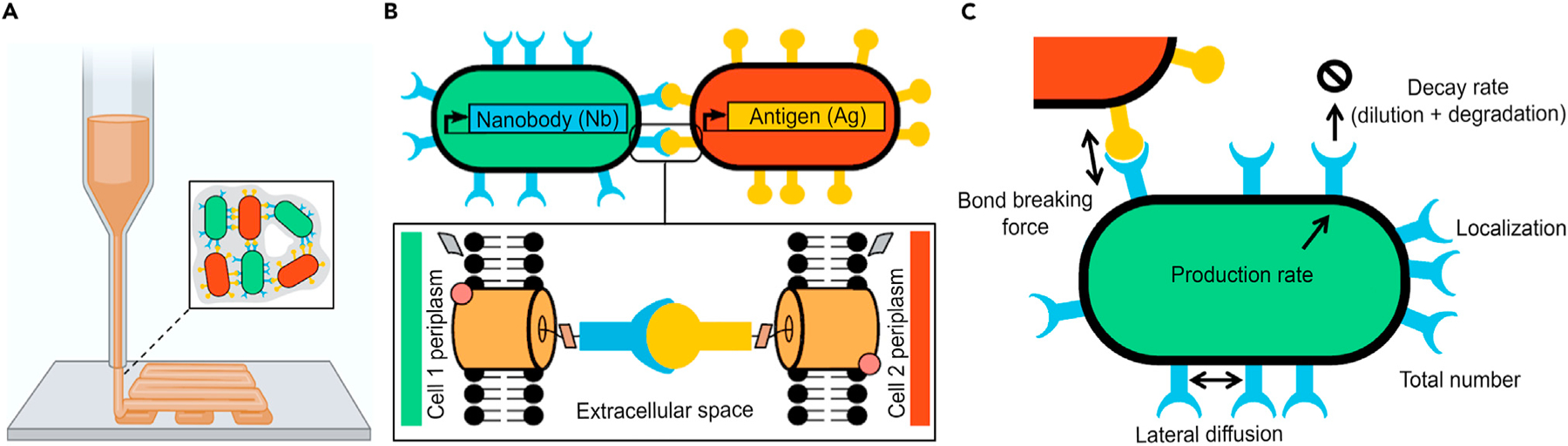
The quantification of the main biophysical parameters for synthetic adhesins is the key for the rational bottom-up engineering of living bacterial materials including the quantitative and predictable tuning of the material properties (A) Schematic of engineered living materials (ELMs) made from *E*. *coli* bacteria mediated by synthetic adhesins with utility, for example, for 3D bioprinting. (B) Schematic of previously engineered synthetic cell-cell adhesin toolbox.^7,14^ (C) Key biophysical parameters of synthetic adhesins in *E*. *coli* are determined (see Table 1 for measured values). Illustrations partially adapted from Glass and Riedel-Kruse.^7^

The emerging discipline of engineered living materials (ELMs) highlights many outstanding properties over traditional materials, and synthetic biology methods are a key up-and-coming approach for ELMs.^1–5^ Cells promise to be versatile and modular material building blocks for ELMs that can harness the near-unlimited complexity and functionality of multicellular systems.^4,6,7^ For example, bacterial synthetic multicellular “consortia” can be used to enable programmable smart materials and artificial tissues including bioprinting (Figure 1A),^1,8^ modularizable pathway engineering platforms for natural drug biosynthesis,^1^ *in vivo* drug-delivery vehicles,^9,10^ and build-to-understand biofilm disease models,^11^ all while being sustainable and biodegradable.^1^

The control of cell-cell adhesion is fundamental for multicellular ELMs, as it enables cells to stick together, allows patterning algorithms (such as self-assembly, differentiation, and potentially synthetic development),^4,6,7,12^ and also determines macroscopic material properties such as viscoelasticity or tensile strength.^1,13^ Previous work established a synthetic cell-cell adhesion toolbox in bacteria consisting of a library of heterophilic nanobody-antigen (Nb-Ag) adhesin pairs that are surface displayed on the outer cell membrane^7,14^ (Figure 1B). This toolbox provides control over adhesion strength and tunability through chemical inducibility, specificity between adhesion pairs, and homophilic cell-cell interactions between cells due to composability.^7^ ELMs at various length scales with interesting properties such as self-growth, programmability, fast recovery under stretching or bending, and open-surface microfluidics have already been demonstrated with this synthetic adhesin toolbox.^7,12,15^ Additionally, other synthetic cell-cell adhesins have been developed in both bacterial and eukaryotic systems.^16–19^

The quantitative and biophysical characterization of such synthetic adhesion toolboxes is significantly lacking,^4–6^ but it is key for the bottom-up prediction of macroscopic material properties from the synthetic building parts, which is also deficient.^4,20^ This is in stark contrast to synthetic biology at the molecular and gene-regulatory level, where, for example, synthetic promoter and repressor systems have been characterized in great detail^21,22^ and successfully used to quantitatively predict the behaviors and functionalities of complex genetic circuits.^23,24^ Synthetic biology pursues the ability to flexibly engineer many-component systems from libraries of standardized and well-characterized parts;^6,25^ consequently, the equivalent characterization of synthetic adhesins for ELMs is key.

This paper presents a general framework for quantifying the key parameters of synthetic adhesins in *Escherichia coli* (Figure 1C) that are crucial for engineering synthetic multicellular materials.^4,6^ Specifically, we measure the synthetic adhesins’ production and decay rate, their density, their spatial distribution and diffusion inside the cell membrane, and their intermolecular bond-breaking force (Figure 1C). For example, the tensile strength and the viscoelasticity^1,13^ of an ELM may depend on whether cells can slide past each other and rearrange their relative position and orientation, which critically depends on the number of adhesins per cell, their stability (longevity), their pairwise binding strength (bond-breaking force), and their ability to move within the cellular membrane. Based on these measured parameters, we then demonstrate how to predict bottom-up and quantitatively tune the macroscopic mechanical properties of an ELM. As a suitable test case, we focus on material tensile strength, which is relevant for bioprinting among other applications (Figure 1A).^26,27^ This generic framework for quantifying synthetic cell adhesin properties will also support basic science and engineering beyond ELMs.

## RESULTS

### Number of adhesins and spatial surface distribution

**Figure 2.**
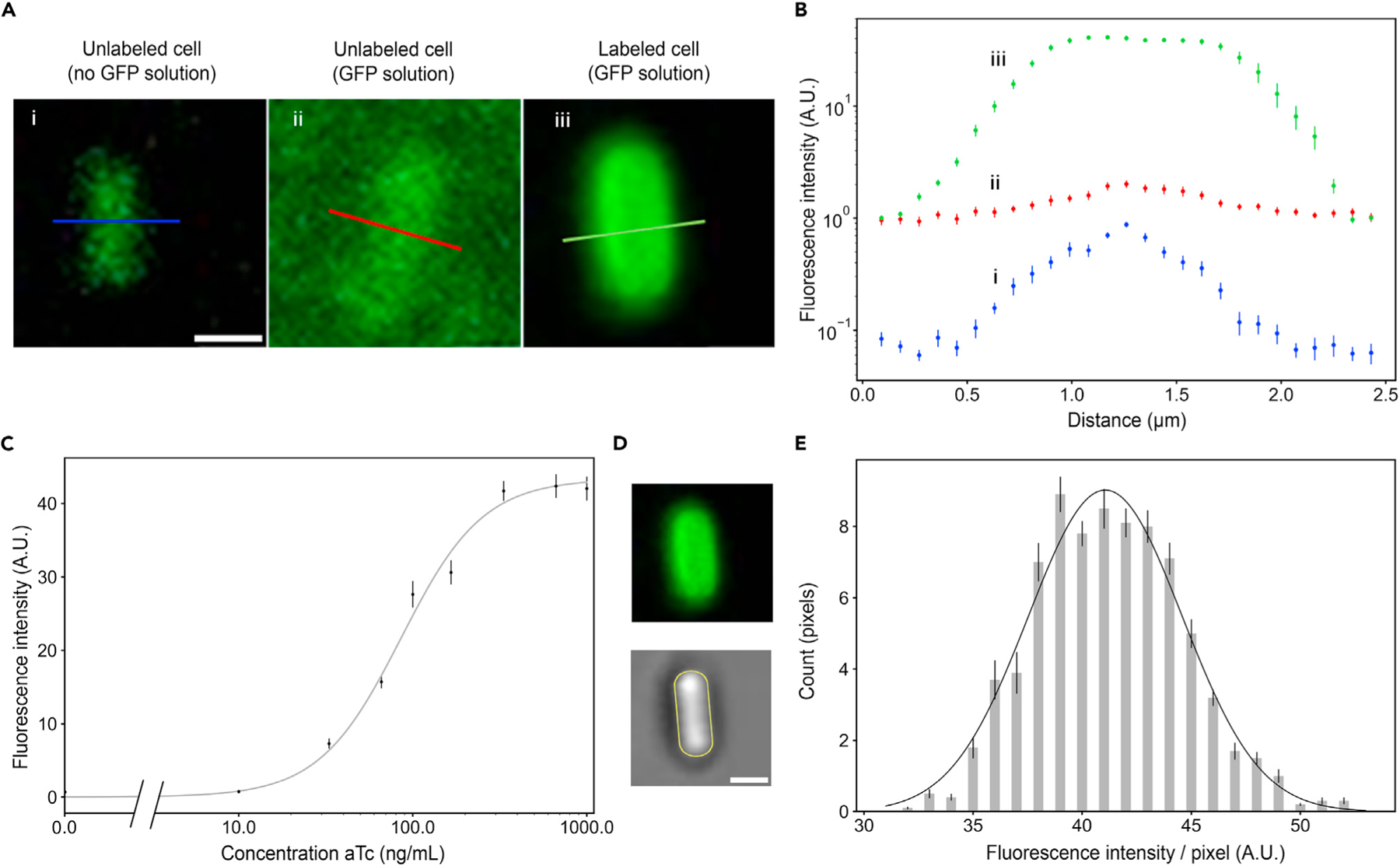
The number of synthetic adhesins on the cell surface, its dependencies on inducer concentration, and its spatial distribution are determined using fluorescent confocal imaging (A) Representative images of GFP Nb-expressing cells imaged under different conditions along with a wild-type cell for comparison (lines indicating the cross-sections along which the intensity was quantified—see B; intensity scaled differently in images for better visual clarity). (B) Fluorescence intensity profiles (log scale) across cells from the varying conditions presented in (A). The peak in the red curve is quantitatively consistent with the autofluorescence artifact observed for the blue curve and is correspondingly corrected for in the model. (C) Fluorescence intensity dependency on inducer concentration. Solid line: fit to a Hill function (Equation 1). (D) Example cell (top) and outline of cell surface (bottom) as analyzed in (E). (E) Fluorescence intensity distribution of pixel values around an anti-GFP cell under maximal level of induction indicating homogeneous adhesion distribution over the cell surface (gray bars), fitted to a normal distribution curve (black line). Data points represent mean ± SEM. Scale bars, 1 µm (A and D).

First, we quantified the number of adhesins per *E*. *coli* cell by expressing Nb against GFP at maximum induction (300 ng/mL anhydrous tetracycline [aTc]) on these cells^7^ and then comparing the fluorescence intensity against a known standard. Specifically, we labeled the cells with GFP and compared the resulting fluorescence intensity per cell with a non-fluorescent cell bathed in a solution of known GFP concentration (365 nM) (Figure 2A and Note S1). We corrected for background and out-of-focus-plane contributions, similar to previous work^28^ (Figure 2B). As 1 nM GFP solution corresponds to 0.6 molecules/*μ*m^3^, the number of adhesin molecules per cell under maximum induction then is $N_{\text{adh}}(300)=15,300\pm 4,100$ (mean ± SEM, henceforth used throughout unless stated otherwise) (n=20 cells). As GFP is only labeling the bacterial surface, this corresponds to a surface density of $\rho_{\text{adh}}=3,400\pm 1,200$ adhesins/μm^2^ given a bacterial cell length and radius of I=1.80±0.25 μm and r=0.40±0.10 μm, respectively (Note S1). At an intermediate induction of 100 ng/mL aTc—as in many of the following experiments—this would then correspond to 7, 300±1, 800 adhesins per cell, or a surface density of 1,700± 600. A potential caveat with this method is that in solution GFP can exist in a bright monomeric and a dark oligomeric form,^29^ which could lead to an overestimation of the adhesin number on the cell surface, as bound GFP are in the monomeric form. Yet our result is in line with previous work that used a western blotting technique^30^ (Note S1).

Next, we determined the dependencies of the total adhesin number on aTc inducer concentration (Figure 2C). We tested aTc concentrations between 0 and 1, 000 ng/mL. We found effective saturation at 300 ng/mL, and we observed unhealthy characteristics at 1, 000 ng/mL (but not below 600 ng/mL), e.g., filamentous growth or lysis (data not shown). This saturation concentration is consistent with prior work on the TetR repressor system.^31,32^ We then fitted the following Hill equation^33,34^ (Note S1 and Figure 2C):

(Equation 1) $$I(C)=f\cdot \frac{N_{\text{adh},\text{max}}}{N_{\text{pix}}}\cdot \frac{C^{\text{n}}}{C_{1/2}^{\text{n}}+C^{\text{n}}},$$

with C being the inducer concentration, $I\left(C\right)$ the corresponding fluorescence intensity per pixel, $N_{\text{pix}}$ the total number of pixels per cell, $C_{1/2}=85.6\pm 6.5\;\text{ng/mL}$ (the inducer concentration for half-maximal expression), n=1.8±0.1 (the Hill coefficient), which is consistent with a cooperativity factor of 2 for aTc,^35^ f=2.7±0.3 being a conversion factor between the adhesin number and fluorescence intensity, and $N_{\text{adh,max}}=17,400\pm 9,600$ molecules/cell (which is close to $N_{\text{adh}}(300)$).

Next, we investigated whether adhesins are homogeneously distributed over the surface or partially localized. We selected cells with both poles in focus and measured the fluorescence intensities along the circumference of the cell, at the poles, and in the middle (Figure 2D). We observed that the fluorescence is normally distributed along this circumference, where the mean and standard deviation of 41 ± 4 a.u./pixel is consistent with the total number of adhesins per cell as determined above (Figure 2E and Note S1). Next, we measured the fluorescence intensity of 10 pixels in the middle and at one of the poles, and we did not observe any significant difference (Student’s t test, p=0.56). Thus, we conclude that the adhesins are homogeneously distributed over the cell surface.

### Kinetics of adhesin turnover

Next, we were interested in characterizing the kinetics of the adhesin number at a given inducer concentration, $N_{\text{adh}}(C)$, due to their effective production rate a(C) and their effective decay rate $b_{\text{eff}}$, with

(Equation 2) $$a(C)=a_{\max}\cdot \frac{C^{\text{n}}}{C_{1/2}^{\text{n}}+C^{\text{n}}}=N_{\text{adh}}(C)\cdot b_{\text{eff}}$$

and where $a_{\max}$ is the maximum protein production rate. Generally, $b_{\text{eff}}$ is the sum of specific degradation rate $b_{\text{deg}}$ due to destruction and the dilution rate $b_{\text{dil}}$ due to cell division.^33^ The specific degradation rate also strongly depends on environmental conditions, for example, growth vs. starvation, where degradation machinery has different activities.^36^

**Figure 3.**
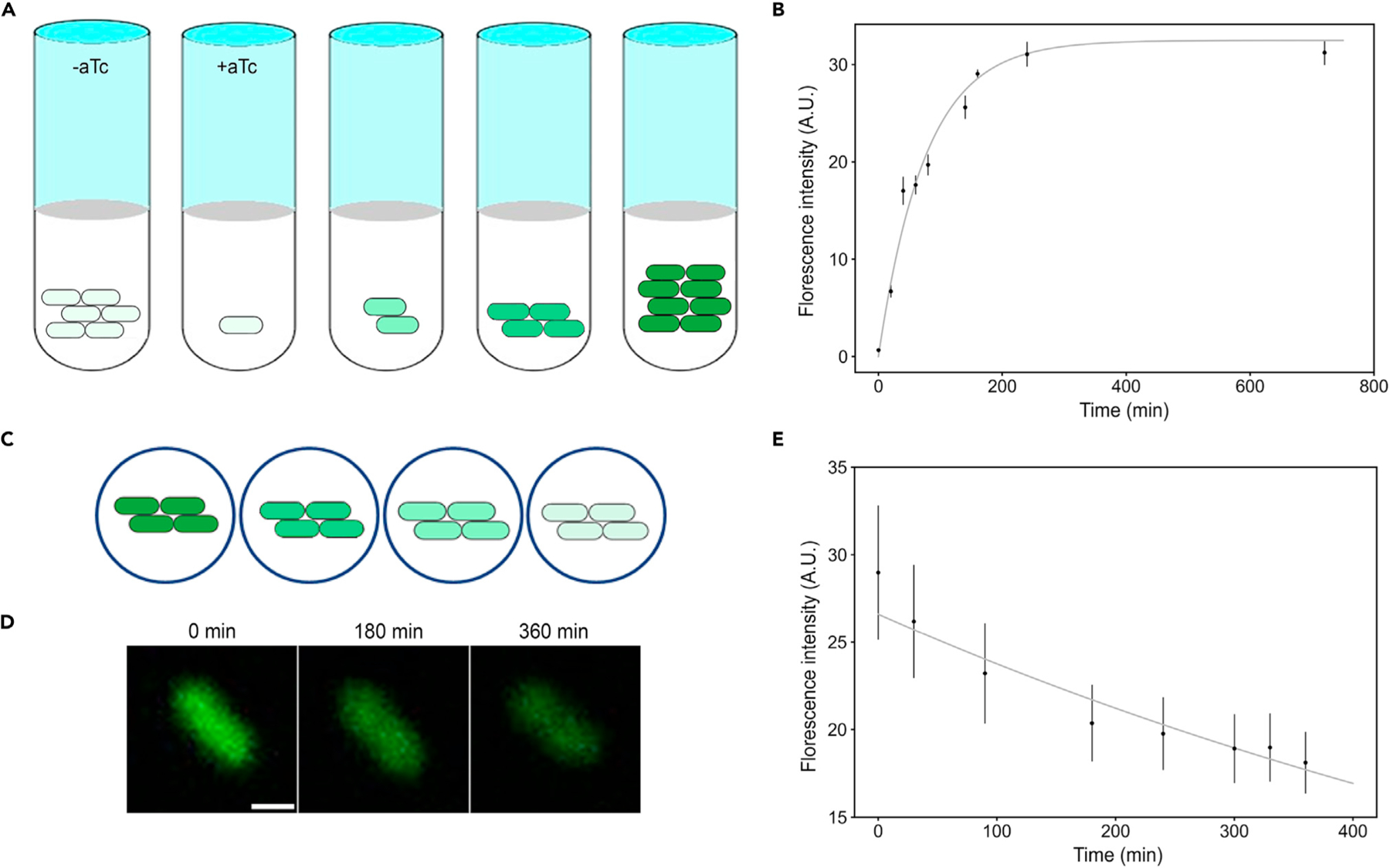
The degradation and production rates of synthetic adhesins are determined through different experimental settings (A) Schematic illustrating the production of adhesins in growing cell cultures where uninduced cells were transferred in medium with aTc. (B) Production curve of the fluorescence intensity of GFP Nbs labeled with GFP (as in A). (C) Schematic illustrating the degradation of adhesins during starvation where induced cells were transferred on agarose pads without nutrients or aTc. (D) Confocal images of the fluorescence intensity of labeled anti-GFP cells over time (as in C). Scale bar, 1 µm. (E) Decay curve of the fluorescence intensity of GFP Nbs labeled with GFP forced to go toward the autofluorescence intensity values (as in D). Data points represent mean ± SEM.

To determine these rates under growth conditions, we set up an overnight culture of uninduced GFP-Nb-expressing bacterial cells, which were later back-diluted for 4 h and then transferred in medium with 100 ng/mL aTc (Figure 3A). We then took samples at different time points t, labeled them with GFP, and quantified adhesin expression with fluorescent microscopy (Figures 3A and 3B). We fitted an exponential growth function for the average intensity per pixel I(t) (Note S2):

(Equation 3) $$I(t)=f\cdot \frac{a_{\max}}{b_{\text{eff}}}\cdot \frac{C^{\text{n}}}{C_{1/2}^{\text{n}}+C^{\text{n}}}\cdot \left(1-e^{-t\cdot b_{\text{eff}}}\right).$$

Using f, $C_{1\text{/}2}$, and n as above, the fit to Equation 3 leads to $a_{\max}=9,900\pm 2,100$ adhesins/cell/h (for example, at 100 ng/mL aTc this corresponds to a(100)=5,600±1,200 adhesins/cell/h, and which is ultimately also promoter and plasmid specific^33^) and $b_{\text{eff}}=0.78\pm 0.12h^{-1}$ (n=6 cells) (Figure 3B). This effective decay rate is not statistically different from the dilution rate due to cell-division rate of $0.82\pm 0.30\;h^{-1}$ (n=8 samples) as we determined from ${\text{OD}}_{600}$ measurements of the cell culture (Note S2, Student’s t test, p=0.6), i.e., dilution dominates over degradation. Both decay and production rates are consistent with literature values in related systems.^33,37^

To measure the specific degradation rate of adhesins during starvation conditions, we induced adhesion expression followed by labeling with GFP, then transferred these cells onto agarose pads without nutrients and without inducer (Figure 3C). Using quantitative fluorescence time-lapse microscopy,^38^ we monitored the average fluorescence intensity per pixel over time (Figure 3D). We fitted an exponential decay function, where now a and $b_{\text{dil}}$ are zero as the cells are not dividing (Figure 3E). We also corrected for potential photofading and active GFP degradation, leading to $b_{\text{deg}}=0.05\pm 0.01\;h^{-1}$ (n=16) (Note S2), which is consistent to degradation rates measured for other *E*. *coli* proteins.^39^ We expect the mechanisms of degradation to include outer membrane proteases^39–41^ or periplasmic proteases,^42^ as has been elucidated for several outer membrane proteins in *E*. *coli*, rather than environmental factors (i.e., extreme temperatures or pH).

### Diffusion coefficient

**Figure 4.**
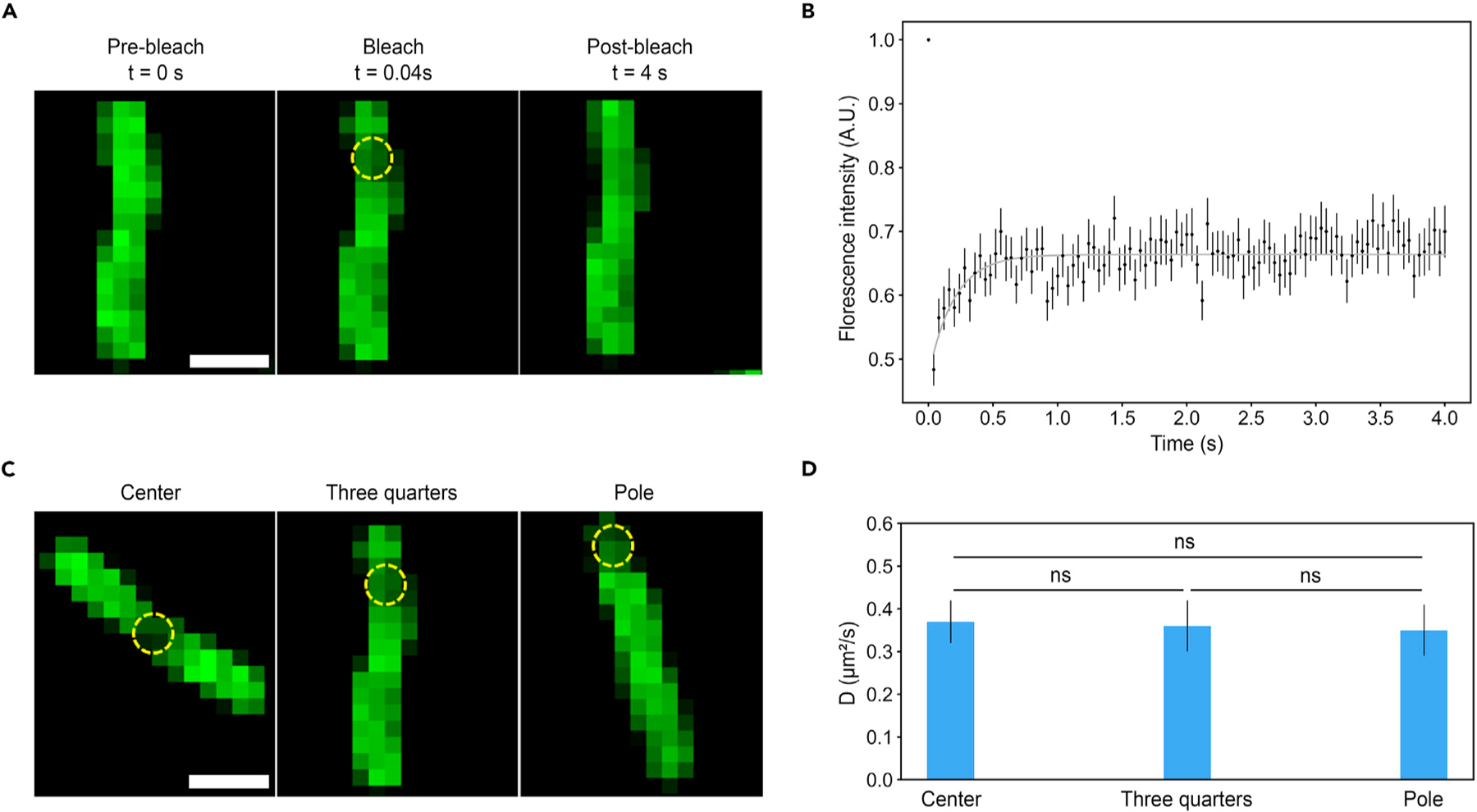
The lateral diffusion coefficient of synthetic adhesins in the cell membrane is determined through FRAP experiments (A) Circular bleached area on labeled GFP Nb-expressing cells. Dotted yellow line represents approximate illustration of the bleached area. (B) The normalized and corrected-for-photofading fluorescence recovery curve of the bleached area. (C) Bleached area in different regions of the cell. (D) Diffusion coefficient corresponding to different areas of the cell. Scale bars, 2 µm (A and C). Data points represent mean ± SEM; ns, not significant.

We determined the lateral diffusion constant of adhesins in the outer cell membrane *in vivo* through fluorescence recovery after photobleaching (FRAP) on a confocal microscope.^43^ First, we chose a bleaching location at three-quarters of the cell length, and we determined the mobile fraction of $M_{\text{f}}=0.37\pm 0.04$ with a diffusion coefficient of $D=0.36\pm 0.06\;\mu {\text{m}}^{2}\text{/s}$ (n=16 cells) (Figures 4A and 4B). We controlled these measurements for various instrument settings (Note S3). Furthermore, these values are consistent with measurements on other outer membrane proteins (OMPs) in *E*. *coli* with typical values between 0.05 and 0.6 *μ*m^2^/s.^44^ We also expected that the adhesin LysM domain for peptidoglycan binding would not affect the diffusion dynamics based on results in previous studies.^45,46^

We next investigated whether this diffusion constant is dependent on the region of the cell. Consequently, we measured diffusion constants at the center, at three-quarters of the cell length, and at the pole of 0.37±0.05 *μ*m^2^/s, 0.36±0.06 *μ*m^2^/ s, and 0.35±0.06 *μ*m^2^/s, respectively (Student’s t test, *p>* 0:5) (Figures 4C and 4D). Note that the raw value at the pole was multiplied by 2 in order to correct for the fact that at this position the fluorescence influx during recovery only proceeds from one direction. Due to OMPs being organized in islands, there might be other diffusion kinetics on significantly different time and length scales^47^ but that are outside of the present scope. We conclude that the lateral diffusion of the adhesin molecules in the cell membrane is the same everywhere on the cell surface.

### Adhesin bond-breaking force

**Figure 5.**
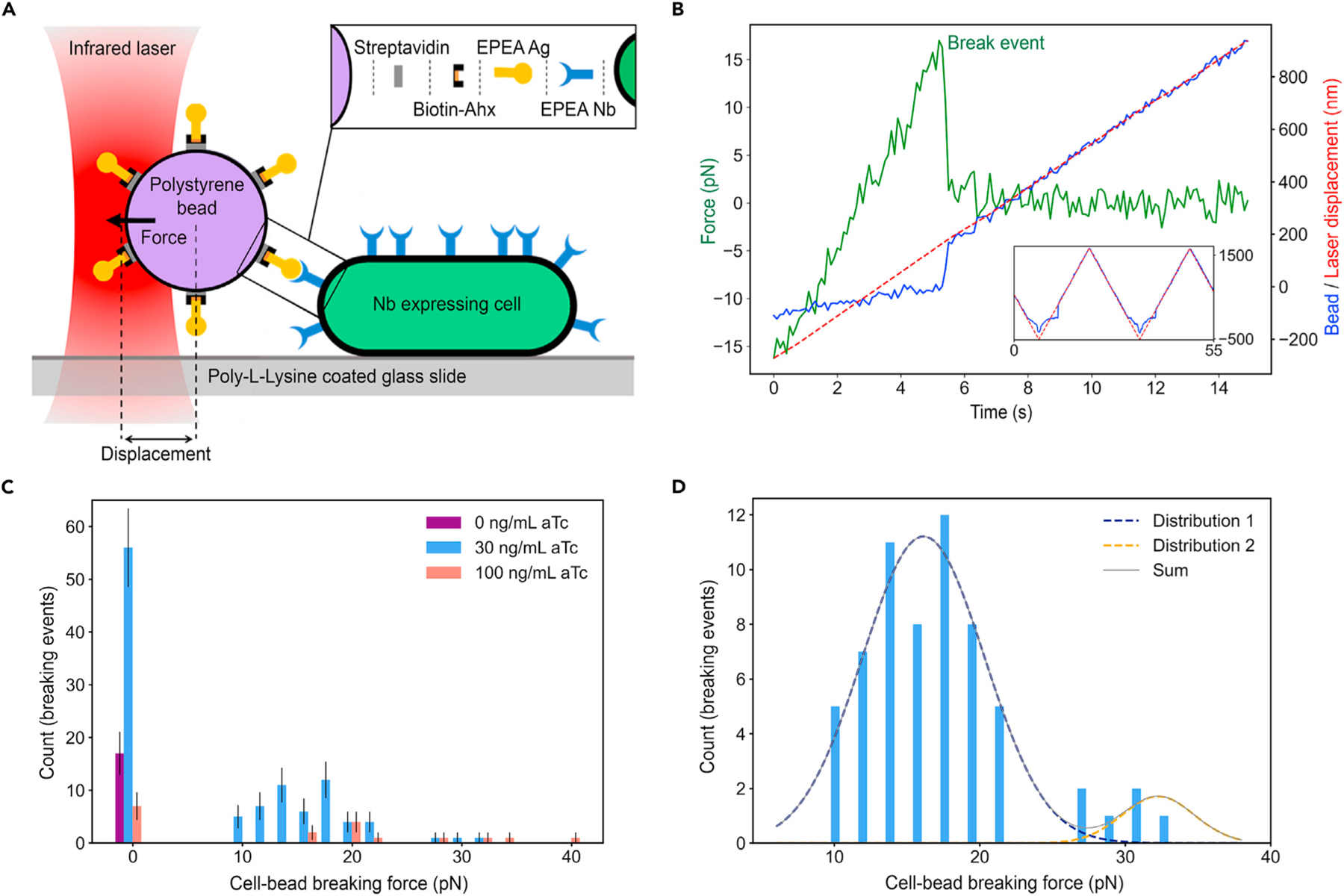
The single adhesin pair bond-breaking force is measured with an optical trap (A) Experimental setup: measuring force between cell attached to glass surface and bead held in optical trap (infrared laser), where trap is oscillating back and forth with frequency f and amplitude A, frequently forming and breaking bonds. Linker length not drawn to same scale as bead and cell. (B) Representative raw data of laser vs. bead displacement over time (red vs. blue curve, respectively) showing individual attaching and breaking events (green curve, difference between blue and red, multiplied by stiffness of the optical trap). (C) Measured breaking forces between the bead and the cell at different concentrations (0 force implies non-binding events). (D) Fitting 30 ng/mL data in (C) to a sum of multiple Gaussian functions for differentiating between single- and multiple-bond-breaking events. Data points represent mean ± SEM.

We used optical trapping experiments to measure the force necessary to break the molecular bond between a pair of complementary Nb-Ag adhesins^48,49^ (Figure 5A). We induced cells to express EPEA Nb (EPEA refers to the 4 amino acids of this small peptide, i.e., glutamic acid, proline, glutamic acid, alanine) and functionalized streptavidin-coated polystyrene beads (diameter 1.7 *μ*m) to present EPEA Ag-linker-biotin-streptavidin on their surface^49,50^ (experimental procedures). We introduced these functionalized beads into a custom-made flow cell and captured an individual bead with the optical trapping laser. We then brought the bead into contact with an *E*. *coli* cell that was unspecifically bound by poly-L-lysine (PLL) to the glass surface of the flow cell. After confirming contact between bead and cell, we oscillated the optical trapping laser back and forth with frequencies and amplitudes in the ranges of f=0.01−0.22 Hz and A=2.2 μm, respectively. This led to frequent binding and subsequent breaking events between the bead and the cell, as recorded by the displacement between bead and laser, which—when multiplied by the calibrated stiffness of the optical trap—reveals the corresponding breaking forces (experimental procedures) (Figure 5B).

Zero breaking force implies that no attachment had formed between bead and cell prior to pulling. For the majority of the 282 trials conducted, we used a loading rate of 20 pN/s; however, we investigated a range of loading rates between 5 pN/s and 100 pN/s, finding no statistically significant variation in Nb-Ag bond rupture force.

We observed a distribution of breaking forces for different levels of aTc induction (Figure 5C). Note that forces above ~40 pN are not considered valid due to equipment limitations, as the force-displacement relationship for the optical trap becomes non-linear, which does not affect reliability of the reported final results (experimental procedures). We did not observe any binding at 0 ng/mL aTc induction, implying that the cell-bead attachment is adhesin specific (Figure 5C). Given the density of potential binding sites observed in fluorescence measurements above, we estimated 30 ng/mL aTc to form between0 and 2 adhesin bonds given the dimensions of the cell, bead, and length of the entire adhesin bond (including linkers) (Figure 2 and Note S4). We then hypothesized that this force distribution (Figure 5C) clusters around multiples of approximately 16 pN, a value that would correspond to the single bond rupturing force. Consequently, we fitted a sum of two Gaussian distributions centered on multiples of an unknown value and obtained a breaking force of $F_{\text{b}}=16.1\pm 0.4\;\text{pN}$ (n=14 cells) (Figure 5D); in contrast, fitting to a single or three Gaussians led to poor fits (Note S4). In addition, we determined that multiple pulling trials on the same cell did not lead to a significant destruction of the adhesins, illustrated by the cell’s ability to keep forming bonds with the bead after several binding/unbinding events (Note S4). We also attempted to determine the binding kinetics $k_{\text{on}}$ and $k_{\text{off}}$, similar to data reported in El-Kirat-Chatel et al.^51^ Given our instrument resolution, we can only provide the lower bound for $k_{\text{on}}$ to be 1.5 M^−1^s^−1^, while $k_{\text{off}}$ could not be distinguished to be different from 0 (Note S4).

Note that different Ag-Nb pairs are expected to have different bond-breaking forces in general. Based on the molecular interactions involved (i.e., electrostatic interactions, hydrogen bonds, van der Waals forces, and hydrophobic interactions), bond-breaking forces are expected to be on the order of tens of pN; for example, atomic force microscopy reveals the rupture force for the Nb-GFP complex *in vitro*^52^ in the range of 28–56 pN, or for natural adhesins, such as cadherins, the rupture varies between 35 and 150 pN.^53^ Furthermore, these values can be modulated by changing the Ag-Nb affinities, for example due to point mutations that determine the structure of the binding sites^54^ or through the development of fusion nanobodies.^55^ Other engineered systems that promote cell-cell binding, such as the Spycatcher-Spytag system with its covalent interactions, present breaking forces above 1 nN.^56^

### Tuning ELM properties: Tensile strength

**Figure 6.**
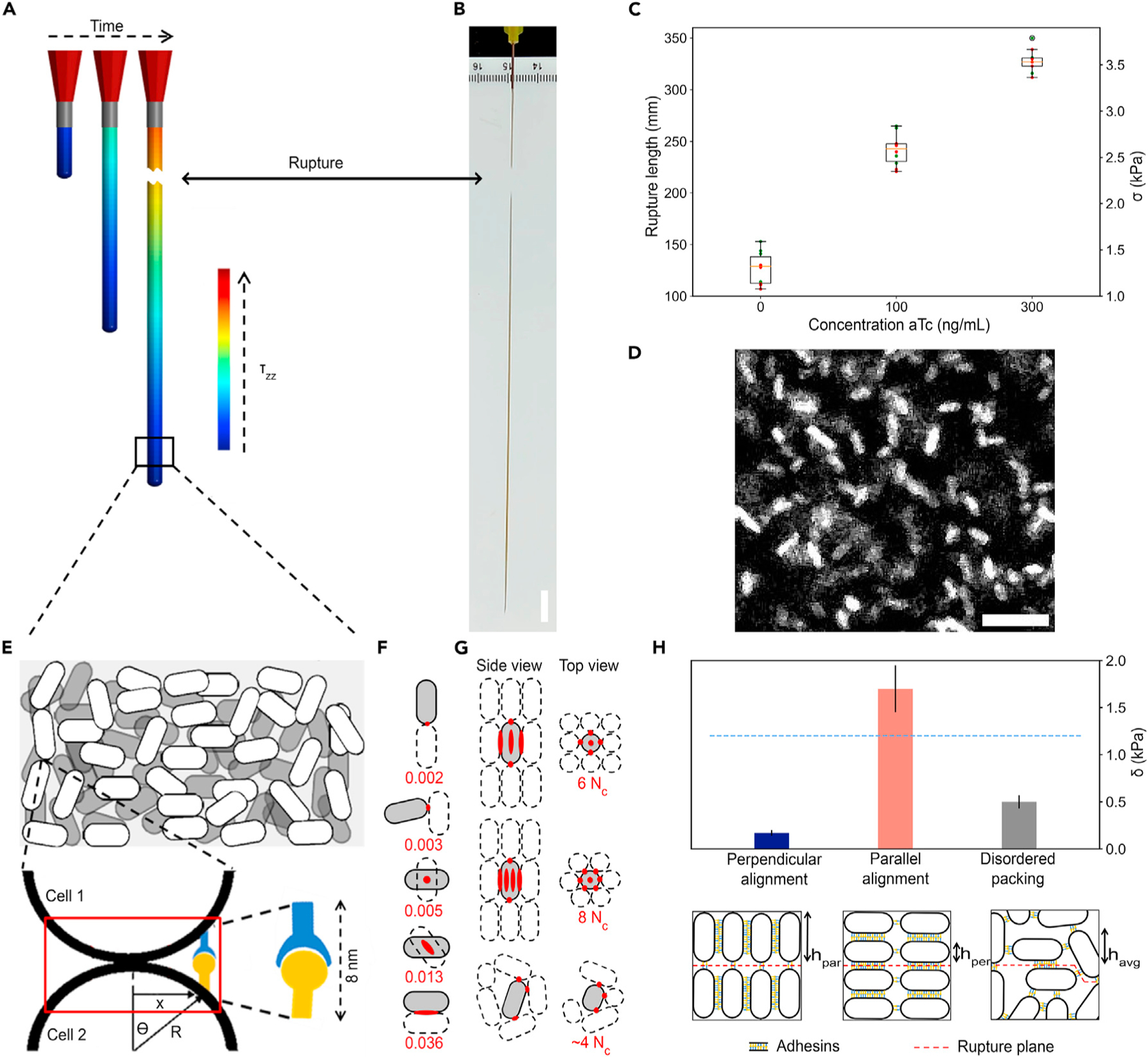
Macroscopic material properties can be quantitatively predicted from the molecular and cellular adhesin properties as demonstrated on tensile strength as an example (A) Schematic of three snapshots in time of an *E*. *coli* cell pellet being extruded from the syringe, where the stress near the syringe increases as the string gets longer. (B) Picture of the material string breaking. (C) The rupture length and corresponding tensile strength, $\sigma_{\exp}(C)$, of the material string depending on different adhesion levels (red and green points: different experimental days; whisker plot, min; lower quartile, median; upper quartile, max; circled green dot, outlier). (D) Confocal image revealing dense packing but no obvious alignment of cells inside the material string (see Note S5). (E) Schematic of presumed disordered dense packing of the extruded material (top), where due to curvature and size of adhesion molecules two cells can only have a small surface area of connection (red, bottom); this area depends on the relative configuration between cells as shown in (F). (F) Examples of different pairwise configurations of two attached *E*. *coli* cells and the corresponding connection area available for synthetic adhesin binding, ${𝓐}_{\text{con}}$(red area, dimensionless, as fraction of total cell surface area; minimal and maximal possible value on top and bottom, respectively). (G) Examples of different possible 3D packings of cells inside the material and the corresponding number of contact points, $N_{\text{c}}$: (from top) primitive tetragonal, hexagonal, disordered packing (many other regular packings are possible, with up to $N_{\text{c}}=12$^59^). Red areas indicate contact points to nearest cells where cell makes physical contact through adhesins. (H) Excess tensile strength of the material string due to adhesion, δ(C), at 100 ng/mL: modeling a primitive tetragonal lattice with the long axis of cells arranged perpendicular (blue) and parallel (orange) to the rupture plane, respectively, and modeling disordered packing similar to illustration in (E) (gray) (ordering indicated by h); experimental results from (C) (blue horizontal line). Data points represent mean ± SEM. Scale bars, 1 cm (B) and 5 µm (D).

We next investigated whether macroscopic material properties could be quantitatively tuned and predicted from these molecular and cellular parameters. We chose tensile strength as a suitable test bed, which is defined as the maximum stress (i.e., pulling force per cross-sectional area) that a material can withstand before breaking. We used the previously developed homophilic cells,^7^ i.e., cells expressing both Ag and Nb adhesin of the same p53TA pair, which leads to a homogeneous material of one cell type and avoids mixing issues when working with the two strains, each just expressing one of the corresponding adhesins^15^ (Note S5). We prepared high-cell-density pellets through centrifugation inside a syringe, which were then extruded through a blunt needle with a syringe pump that was kept at a constant flow rate. The extruded material string eventually ruptures under its own weight (Figures 6A and 6B).

This rupture point allowed us to experimentally determine the tensile strength $\sigma_{\exp}=mg\text{/}A_{\text{s}}=\rho l_{s}g$, with the gravitational force mg, the cross-sectional area $A_{\text{s}}$, the length $I_{\text{s}}$, and the volume $V_{\text{s}}=I_{\text{s}}A_{\text{s}}$ of extruded material string below the breaking point, the material density $\rho =m\text{/}V_{\text{s}}=1,100\pm 50\;{\text{kg/m}}^{3}$, and the standard gravity *g* = 9.81 m/s^2^. This then led to tensile strengths $\sigma_{\exp}(C)$ in the range of to 1.37 – 3.53 kPa, depending on induction level C (Figure 6C). The fact that the tensile strength at zero induction level, $\sigma_{\exp}(0)$, is not zero can be understood from other (potentially non-specific) adhesive interactions between cells.^57^ The difference in tensile strength between non-induced and induced cells of $\delta_{\exp}(C)=\sigma_{\exp}(C)-\sigma_{\exp}(0)$ is then due to synthetic adhesin pairs. Under the present experimental conditions, this “excess tensile strength” $\delta_{\exp}(C)$ also appears to be proportional to the synthetic adhesin expression level according to Equation 1 (Figure 6C and Note S5). We thus observed a systematic and significant increase in string length and, hence, tensile strength between non-induced, partially induced, and fully induced adhesin levels (Figure 6C), highlighting the tunability of this material property.

### Quantitative bottom-up material property predictions

Finally, we investigated whether this excess tensile strength, δ(C), could be predicted from first principles and quantitatively based on the earlier measured molecular and cellular parameters. δ(C) depends on the number of adhesin bonds between neighboring cells that need to rupture, which in turn depends on the area of contact between any two cells as well as the overall ordering of cell packing. We performed confocal imaging of the material string to investigate how cells are packed and ordered after having been extruded. We observed dense packing, i.e., a solid-to-volume fraction of $f_{\text{sv}}=0.5$ (also confirmed by ${\text{OD}}_{600}$ measurements), which is close to the theoretical maximum, yet we did not detect any significant alignment of cells based on the circular mean analysis (Figure 6D and Note S5). We therefore conclude that the cell packing is dense but largely disordered, likely similar to a compact disordered spherocylinder packing as was modeled previously^58^ (Note S5).

For the material string to rupture under its own weight, all cell-cell contacts in one cross-sectional area need to break apart. Due to the curvature of the cells and the adhesin pair length of 2M=8 nm, only adhesins within a certain contact area can actually contribute to the adhesion between any two cells (Figure 6E and Note S5). We correspondingly define the fraction of the total cell area, ${𝓐}_{\text{con}}$(dimensionless), that is committed to this cell-cell contact and needs to break apart. ${𝓐}_{\text{con}}$ depends on how the two cells are placed relative to each other, varying between 0.002 and 0.036 (Figure 6F and Note S5). Furthermore, any cell could have one or multiple such contact areas that need to break apart. This number depends on packing arrangement and, thereby, the total number of nearest neighbors $N_{\text{c}}$ that any cell has physical contact with, and where many packing arrangements are possible (Figure 6G). Here we consider for simplicity only the ordered packing of a primitive tetragonal lattice,^59^ with $N_{\text{c}}=6$, as well as disordered packing, with $N_{\text{c}}\sim 4$ (Figure 6G and Note S5). In these cases, only about one such contact area needs to break per cell pair, and no additional correction factor is required in the following derivation (Figures 6F and 6G; Note S5).

The number of adhesins pairs that need to break per cell is then the product of this area ${𝓐}_{\text{con}}$ (Figure 6F) and the total number of adhesins per cell $N_{\text{adh}}(C)$ at a given inducer concentration C (Note S5). The homophilic cells encode the Nb and Ag adhesins on a medium- and low-copy-number plasmid with 20–30 and 10–12 copies, respectively.^60^ Hence there are about half as many Ag than Nb adhesins, and the Ag adhesins will be the limiting factor for the number of pairwise connections between any two cells that can be formed. We therefore reduce $N_{\text{adh}}(C)$ as determined above for the medium-copy-number plasmid (Figure 2) by a correction factor of $f_{\text{p}}=0.5$. For C=100 ng/mL aTc induction, this leads to the average number of adhesin pairs connecting any two cells $\left(f_{\text{p}}\cdot N_{\text{adh}}(C)\cdot {𝓐}_{\text{con}}\right)$ of 7±2 and 130±30 for perfect vertical and horizontal packing, respectively (Figure 6G); for the experimentally observed disordered packing, we then estimate an intermediate value of 30±5 (Note S5).

The total number of cells involved in the tensile rupture event also depends on the solid-to-volume fraction of cells to the string volume, $f_{\text{sv}}$, the cell volume, $V_{\text{cell}}$, and again how cells are ordered, connected to each other, and arranged relative to the rupture plane. Here h is the height of a cell layer (Figure 6H), with $V_{\text{cell}}=h$ being the effective area a cell takes up in the rupture plane, thereby determining how many cells can be packed into this plane, and with $h_{\text{per}}=I$ and $h_{\text{par}}=2r$ for the highly ordered cases (Figure 6H and Note S5). Ultimately, $N_{\text{cell}}$ is proportional to the cross-sectional area of the material string, $A_{\text{s}}$, and therefore does not explicitly depend on $A_{\text{s}}$ (Note S5). The predicted excess tensile strength of the material due to the synthetic adhesins then is given by

(Equation 4) $$\delta_{\text{model}}(C)=f_{\text{p}}\cdot N_{\text{adh}}(C)\cdot {𝓐}_{\text{con}}\cdot F_{\text{b}}\cdot f_{\text{sv}}\cdot h\text{/}V_{\text{cell}}\cdot$$

Based on these considerations, we then compared theoretical and experimental excess tensile strength at C=100 ng/mL aTc induction. For the two extreme cell configurations (Figure 6H, orange and dark blue bars), i.e., where cells are perfectly aligned and packed perpendicular or parallel to the rupture plane, respectively, we determined $\delta_{\text{per}}=0.17\pm 0.03\;\text{kPa}$ and $\delta_{\text{par}}=1.70\pm 0.25\;\text{kPa}$ (errors determined from uncertainties in the determined biophysical parameters). This also implies that such highly ordered material would be highly anisotrophic.^61^ In a more realistic disordered packing scenario based on our experimental data and analysis (Figure 6D and Note S5), the cell pellet likely consists of some combination of all possible cell configurations (Figure 6F), leading to an intermediate excess tensile strength of $\delta_{\text{avg}}=0.50\pm 0.07\;\text{kPa}$ (Figure 6H [gray bar] and Note S5). These quantitative bottom-up predictions potentially have limitations, such as: (1) material thinning before breaking might lead to a smaller cross-section; (2) additional macroscopic material or ordering defects^62^ might exist; or (3) adhesin diffusion might lead to more adhesion pairs per contact point. Nevertheless, when comparing with our experimental results, we find agreement approximately within a factor of ~3 for the disordered model scenario (Figure 6C), being within the bounds of the two highly ordered, anisotropic model extremes (Figure 6H); agreement also holds true for induction level of 300 ng/mL (Note S5).

## DISCUSSION

In summary, we demonstrated how to measure the key biophysical parameters of synthetic adhesins using a bacterial Nb-Ag toolbox as an example,^7^ and how to then quantitatively predict and tune the macroscopic properties of materials engineered with these adhesins. All measured parameters are summarized in Table 1 and Figure 1C, and the specific values will vary based on the specific synthetic adhesin pairs, plasmids, and promoters used.^7,14,33^ Existing measurements or estimations in related systems (Table 1) provide additional confidence in our findings^30,39,44,52^ (Figures 2, 3, 4, and 5). The molecular bond-breaking force can also be directly related to the molecular $K_{\text{D}}$.^51^ Notably, we did not see variations in diffusivity and localization of the adhesins at the cell poles. In this study, we used three different Nb-Ag pairs, i.e., anti-GFP/GFP (Figures 2, 3, and 4), anti-EPEA/EPEA (Figure 5), and anti-p53TA/p53TA (Figure 6), where the latter two correspond to adhesin pairs 2 and 3, respectively, in Glass and Riedel-Kruse.^7,63^ Each of these adhesin pairs have properties that made them particularly suitable for the type of experiments, i.e., fluorescent labeling via GFP, availability of the Ag as easily modifiable peptide, and the absence of *cis* interactions on the same cell, respectively. We provided sufficient details and controls that should enable others to perform similar characterizations on existing (synthetic) adhesins^7,14,16–18^ as well as newly engineered ones.^63^

Our results revealed multiple surprises regarding how cell-cell adhesins can determine and tune the properties of synthetic as well as natural multicellular bacterial systems. We found that only a very small fraction (~ 1%) and small absolute number of adhesins actually mediate a connection between any two cells, for example, ~ 1 and ~ 30 pairs at 30 and 100 ng/mL induction, respectively (Note S4 and Figure 6). Given their specific and rigid cell shape, each cell has adhesin-mediated contacts with only ~ 4 neighboring cells inside a disordered material, while for highly ordered packing this number can be much higher (Figure 6G and Note S5). Hence, even if initially bound adhesins should become damaged during cell-cell separation, the cellular binding abilities and strengths would stay essentially unaltered over many cellular binding-unbinding cycles, affecting material-level properties such as viscosity.

We also investigated the viability of the adhesin-expressing cells over time under stressful conditions, as well as the stability and capacity to self-regenerate the ELM (Note S6). We previously demonstrated that these synthetic adhesins are compatible with cell growth and division and that they do not affect cell viability.^7^ For further validation, we now additionally monitored the stability of the ELM over time in buffer without nutrients and observed that the material consisting of adhesin-expressing cells was significantly improved compared with non-adhesive cells (Note S6). We also determined that the material degradation timescale is within a factor of 3 compared to the degradation of adhesins (Note S6). In addition, we explored the capacity of the material to self-regenerate and determined that even after several days of exposure to stressful environments (e.g., nutrient deprivation or waterevaporation), the cells were still viable and led to culture growth with no significant difference compared to wild-type cells grown under normal conditions (Note S6).

Many opportunities exist for tunability from the molecular adhesin level to the macroscopic material level, well beyond tuning via gene induction level, as demonstrated here (Figure 6C). For example, binding forces could be lowered by changing the Nb coding sequence responsible for the binding specificity,^18,63^ and the number of adhesins binding between cell pairs could be increased by using longer linkers between membrane and adhesin domain (Figure 6E), by changes in the membrane mobility, or by changing the aspect ratio of cells.^7^ Control over the structural order of cells inside the material should enable significant tuning of material properties including anisotropy, as is known from classic inorganic materials^59,61^ (Figure 6H).

The capabilities of synthetic adhesins enable many stand-out features of ELMs as investigated and demonstrated by multiple research groups. For instance, a programmable living material assembly utilizing bacterial adhesion was demonstrated in wearable sensors to detect bioelectrical signals, whereby these sensors showed self-healing within minutes after stretching.^15^ Adhesin-expressing cells can also be integrated in ELMs capable of responding to environmental cues. This property was explored by several researchers, for example in the development of living biofilm-based materials for mercury bioremediation^64^ or engineered living glue capable of autonomous self-repair.^65^ These applications showcase the potential of adhesin-expressing cells in designing ELMs, where quantitative understanding and tuning of the adhesin properties, as demonstrated here, will further support these developments.

Finally, we note that the analytical and quantitative bottom-up prediction of material properties as demonstrated here for tensile strength (Figure 6) is still rare and challenging for many ELMs and specific properties.^4,6,66^ Theories that do exist often do not provide a reasonable agreement giving non-matching assumptions.^67,68^ Our data and model indicate that the material-level excess tensile strength under the presented experimental conditions is simply the product of molecular adhesin bond-breaking force and number of adhesin pairs between neighboring cells, and that cell dimension, cell geometry, and packing order have a fundamental impact (Figures 6E–6H). This bottom-up predictive power (Figure 6H) will also enable new top-down approaches for measuring molecular parameters, for example, deducing the molecular adhesin binding force from the material’s tensile strength. We expect that mechanistic bottom-up theories for many complex living materials might be challenging to develop and test, and instead pragmatic finite element simulations and machine-learning methods with high predictive power yet fewer mechanistic insights will play a significant role in future materials engineering.^3,4^ Such experimental and theoretical advancements are key for engineered living materials across different fields, for example, bioprinting of living sensors^15^ or therapeutics.^69^ Much of the outstanding work on ELMs^2–4,20^ has not yet been paired with quantitative bottom-up predictions or rheological and biological characteristics^70^; we hope that our results will stimulate more combined experimental-theoretical work of diverse natural and engineered living materials.

## EXPERIMENTAL PROCEDURES

### Resource availability

#### Lead contact

Further information and requests for resources should be directed to the lead contact, Ingmar H. Riedel-Kruse (ingmar@arizona.edu).

#### Materials availability

This study did not generate new unique reagents.

#### Data and code availability

All analyzed data are available in the manuscript or the supplemental information. Raw data and modeling scripts are available upon reasonable request.

### Plasmids and strains

The MG1655 *E*. *coli* strain obtained from the *E*. *coli* Genetic Stock Center (CGSC 6300) was used for all experiments in this study. Plasmids were transformed into chemically competent cells following standard protocol.^7^ Plasmids were sourced from our earlier work,^7^ and the copy number was indicated by previously published literature^60^: pDSG339 (pSB3K3_TetR_pTet_Neae2v1_antiGFP) (GenBank: MH_492430), pDSG320 (pSB3K3_TetR_pTet_Neae2v1_antiEPEA) (GenBank: MH_492391), and pDSG321 (pSB3K3_TetR_pTet_Neae2v1_antiP53TA) (GenBank: MH_492393) are medium-copy-number plasmids (20–30), whereas pDSG288 (pSB4A3_TetR_pTet_Neae2v1_P53TA) (GenBank: MH_492378) is a low-copy-number plasmid (10–12).

### Number of adhesins and spatial surface distribution

Anti-GFP-expressing *E. coli* cells were aerobically grown overnight at 37°C in Luria-Bertani (LB) medium supplemented with kanamycin. Membrane adhesin expression was induced by adding different concentrations of aTc. Both the induced cells and the wild-type control were harvested by centrifugation at 3,000 rpm for 5 min and resuspended in a PBS solution with 0.5% BSA for surface treatment. GFP was added to the bacterial cells and left to incubate for 30 min, then spun down and washed with 0.5% BSA/0.1 M PBS solution three times. The cells were then added to a solution of GFP of known concentration, c=065 nM. Four microliters of cell culture was spotted on a glass slide.

Confocal fluorescence images were recorded with a Zeiss 700LSM laser scanning confocal microscope equipped with a 10-mW laser. The 488-nm laser line was selected for GFP excitation. The sample was visualized with a 63× oil-immersion objective lens (numerical aperture [NA], 1.4), and the pinhole was opened to 1 µm. Images were recorded by scanning the laser over a 16.8 × 16.8 mm field of view. Images were 188 × 188 pixels, with a scan speed of 17.2 µs per pixel and averaged from two successive scans. Laser intensity during image acquisition was maintained at 2% to minimize photobleaching.

### Kinetics of adhesin turnover

To determine the adhesin production rate, the anti-GFP-expressing *E. coli* cells were grown under the same conditions as before except that adhesin expression was not induced. After 24 h, the bacterial cell culture was back diluted 1:1,000 and incubated for another 4 h to reach the exponential growth phase. They were then harvested by centrifugation at 3,000 rpm for 5 min and transferred to LB medium with 0.1 μg/mL aTc. Samples of 100 μL were then collected every 20 min and spun down, followed by the cells being resuspended in a PBS solution to which recombinant GFP was added. The incubation period was 30 min, after which the cells were washed three times with PBS. Four microliters of cell culture was spotted on a glass slide. For the degradation-rate experiment, the membrane adhesin expression was induced by adding 0.1 μg/mL aTc. The overnight culture was back-diluted and induced and after 4 h, the cells in exponential growth phase were harvested by centrifugation at 3,000 rpm for 5 min, then incubated with recombinant GFP for 30 min, washed three times with a PBS solution at the same centrifugal settings as above, and transferred to an agarose pad^38^ made of PBS and lacking inducer. Two microliters of cells was spotted on the agarose pad.

Confocal images were recorded by scanning the laser over a field of view that was typically 20.3 × 20.3 μm (224 × 224 pixels). Images were averaged from two successive scans. Laser intensity during image acquisition was maintained at 2% and the pinhole to 1 a.u.

### Diffusion coefficient

Anti-GFP cells were grown as presented above. Membrane adhesin expression was induced by adding aTc. Prior to measurements, the culture was diluted approximately 1:1,000 into the same medium and grown at 37°C under constant shaking for 4 h. Cephalexin was added to 30 g/mL and the cells were grown for a further 45 min. Cells were harvested by centrifugation at 2,000 rpm (to prevent breakage of the elongated cells) for 5 min and resuspended in a PBS solution. Recombinant GFP was then added to the bacterial cells and left to incubate for 30 min, then washed three times with PBS. Four microliters of cell culture was spotted between a glass slide and a coverslip. During the entire experiment, the stage was heated to 25°C using a climate chamber.

The confocal images were acquired using a 63× oil-immersion objective lens (NA, 1.4) under an incubation temperature of 37°C. Images were recorded by scanning the laser over a field of view that was typically 16.9 × 16.9 μm. The resolution of the images was kept low at 32 × 32 pixels, with a scan speed of 16 ms per pixel to minimize photobleaching. Laser intensity during image acquisition was maintained at 2%. A series of ten prebleach images were acquired, after which the laser was pulsed once at a selected region of the cell with 100% intensity to bleach the area but at a lower scan speed to ensure sufficient bleaching. Postbleach images were recorded at 40-ms intervals for a total of 5 s.

### Adhesin bond-breaking force

Anti-EPEA-expressing *E. coli* cells were grown overnight, and adhesin expression was induced by adding different concentrations of aTc. The overnight culture was back diluted and induced, and after 4 h, cells were harvested by centrifugation at 3,000 rpm for 5 min. The cells were then resuspended 1:10 in PBS. Biotin-Ahx-EPEA peptide was synthesized by GenScript at >95% purity (Ahx: aminohexanoic acid linker used to create extra space between the biotin and the protein for efficient access of biotin-binding entities^50^). The lyophilized peptides were resuspended in water, and their concentration was quantified on a NanoDrop One using the A205/31 method. The biotinylated protein was then attached to the streptavidin-coated beads, purchased from Spherotech (catalog no. SVP-15-5), according to the protocol presented in the TechNote 101 by Bangs Laboratories. The concentration of protein added to the microspheres was ensured to be in excess such that the binding capacity of the beads reached the maximum. The final solution of protein-coated beads was at a concentration of 0.5 mg/mL.

A flow chamber consisted of a glass slide presenting two holes connected to plastic tubing with the inner diameter of 0.02 inches and a 24 × 50-mm coverslip, attached together by double-sided tape such that the height of the chamber was 0.1 mm. PLL was injected into the chamber through one of the tubes and left to dry overnight. The cell-PBS solution was then introduced into the flow chamber at 0.01 mL/min until it was filled and then left to incubate for 30 min. Thereafter, the unbound cells were washed using a PBS solution at a flow rate of 0.01 mL/min. Prior to experiments, the bead solution was diluted 1:20 in PBS and injected into the chamber.

The optical trap was custom built^48^ on an inverted Nikon Eclipse TE200 microscope with a Nikon 100×, 1.4 NA oil-immersion objective and a 10 W Nd:YAG 1,064-nm infrared trapping laser. The trapping beam was steered using an IntraAction Acousto-optic Deflector (AOD). The driving voltage frequency was controlled by an IntraAction frequency board in which the frequency was controlled by custom-written LabView software operated from a Windows 10 operating system. Imaging of the bead and bacteria was completed using a C7300 Hamamatsu camera using custom-written LabView software. The protein-coated microsphere was captured using the optical trap with the laser moving back and forth using an AOD and a custom-written LabView code. The laser frequency of oscillation of $\nu_{\text{laser}}∼0.01\to 0.22\;\text{Hz}$ and a spatial amplitude, A, of oscillation were tuned to ensure contact between bead and *E. coli* and subsequent bond breakage (A was typically between 2.2 and 2.3 µm). For calibration of the trapping laser spring constant, a spherical polystyrene bead was held at the focal point of the trapping laser while calibration was performed. Using custom-written LabView software, the trapping stiffness, $k_{\text{las}}$, was measured by two methods: equipartition theorem and power spectrum analysis.^48^ To obtain this information, a low-power green laser aligned with the trapping laser was used in combination with a Thor Labs Photo-Quadrant Diode (PQD). The calibration laser was focused down to the back focal plane of the objective in which the PQD reads a voltage level associated with the displacement of the trapped bead. Because the trapping stiffness scales linearly with laser power level, to determine the trapping stiffness at full power, a lower power (and therefore lower $k_{\text{las}}$) is typically used^49^ in conjunction with equipartition and power spectrum analyses. The measured $k_{\text{las}}$ was then scaled linearly along with the laser’s power level to achieve the desired final trapping stiffness. For the spectral density measurements, the displacement of the calibration bead was transformed into a power spectral density in which the cutoff frequency was used to determine optical trap stiffness.

The flow chambers containing anti-EPEA cells and EPEA-coated microspheres were mounted onto to the stage of the optical trap. Manually manipulating the stage, a bead was caught at a distance of approximately 4 µm above the glass coverslip (i.e., above the bottom of the slide). This bead was then positioned near an *E. coli* cell that had been adhered to the coverslip. The bead was then lowered to the glass coverslip until contact was made, indicated by observing the bead leaving the focal plane. The bead was then raised away from the coverslip by approximately 200 nm. The position of the stage was then held constant, and the trapping beam was moved by varying the AOD input acoustic frequency $\left(\nu_{\text{aod}}\right)$ so that the bead moved laterally back and forth in a triangular wave pattern at a constant frequency $\left(\nu_{\text{laser}}\right)$. The position of the stage was adjusted so that the bead made contact with the bacterium at the peak of the triangular wave pattern. As the laser is moved away from the bacterium, if the bead has attached to the bacterium, the bead will start to become horizontally displaced from the focal point of the trapping laser. We conducted 282 individual pull force trials, moving the bead perpendicular to the long axis of the bacterium. Typically, the force-displacement curve of the optical trap is linear; however, in our setup, at values above 400 nm from the trap equilibrium (40 pN with our laser power), the force-displacement relationship becomes non-linear. Therefore, forces above 40 pN (i.e., corresponding to more than two adhesin pairs being ruptured) are not included in further analysis so as not to skew the final results. Images and AOD input frequency from each trial were analyzed using custom-written MATLAB code in order to correlate the center of the polystyrene bead to the actual location of the trapping laser. Image analysis was performed using custom-written code in MATLAB. The difference in location between the laser focal point and bead, Δs, was extracted from the data. The rupture-strength force was then computed, $F_{\text{rup}}=k_{\text{las}}\times \Delta s$, where $F_{\text{rup}}$ is the peptide rupture force.

### Tuning ELM properties: Tensile strength

A preculture (3 mL) of homophilic p53 TA *E. coli* strain was grown overnight. Next day, the preculture was transferred to 500 mL of LB supplemented with the corresponding antibiotics and after 4 h of incubation, the adhesin expression was induced by adding aTc. Twenty-four hours after induction, the cells were harvested by centrifugation at 4,000 × *g* for 10 min. The supernatant was then removed and the cell pellet transferred in a 10-mL syringe. After sealing the syringe tip and fixing the plunger in place, the cell pellet was centrifuged again at 4,000 × *g* for 8 min to remove air bubbles. To extrude the material from the syringe, a syringe pump was turned sideways and the flow was set to 0.2 mL/min. A blunt syringe tip with a diameter of 0.8 mm was attached to the syringe tip.

## Supplementary Material

MMC1

## Figures and Tables

**Table 1.** Summary of key biophysical parameters of the synthetic adhesion toolbox

Parameter	Symbol	Unit	Mean	Random error	Systematic error	Expectation	Reference
Total number	$N_{\text{adh}}(100)$	molecules/cell	7,300	1,800	1,500	6,000–8,000^a^	Salema et al.^30^
Diffusion rate	* D *	*µ*m^2^/s	0.36	0.06	0.05	0.05–0.6	Spector et al.^44^
Production rate	a(100)	molecules/cell/h	5,600	1,200	900	6,000^a^	Kalisky et al.^37^
Degradation rate	$b_{\deg}$	1/h	0.05	0.01	0.01	0.13	Maurizi^39^
Dilution rate	$b_{\text{dil}}$	1/h	0.82	0.3	0.1	1.8	Alon^33^
Bond-breaking force	$F_{\text{b}}$	pN	16.2	0.4	1.8	28–45	Klamecka et al.^52^

See also Equations 1, 2, and 3 for definitions;$C_{1/2}=85.6\pm 6.5\;\text{ng/mL}$, n=1.8±0.1, $N_{\text{adh,max}}=17,400\pm 9,600$ molecules/cell, and $a_{\max}=9,900\pm 2,100$ molecules/cell/h. Parameters will vary for specific adhesin pairs (especially $F_{\text{b}}$) and genetic inducers; $N_{\text{adh}}$ and a are stated for 100 ng/mL aTc.

a Regarding comparison to literature, a different inducer (and, hence, likely effective different induction levels) was used.

## Highlights

Synthetic bacterial systems hold promise for medical and smart biomaterials

The study demonstrates quantitative characterization of a synthetic adhesion toolbox

Macroscopic material properties can be tuned and predicted through a bottom-up approach

## PROGRESS AND POTENTIAL

Synthetic multicellular systems provide versatile building blocks for engineered living materials (ELMs). Precise control of cell-cell adhesion is crucial for multicellular material engineering, especially with synthetic adhesion tools becoming available recently. Here, we demonstrate a general framework for quantitatively characterizing the key biophysical parameters of such an adhesion toolbox. We then demonstrate how these parameters allow the prediction and tuning of macroscopic bacteria-based ELM properties such as tensile strength. These results have direct relevance for bioprinting, where the ideal bioink should possess well-defined mechanical and biological characteristics. More broadly, this work bridges synthetic biology and material science and introduces mathematical prediction and rational tuning of ELM properties for aiding quantitative, model-supported ELM development.

## References

[1] An B, Wang Y, Huang Y, Wang X, Liu Y, Xun D, Church GM, Dai Z, Yi X, Tang T-C, and Zhong C (2023). Engineered living materials for sustainability. Chem. Rev 123, 2349–2419. 10.1021/acs.chemrev.2c00512. 36512650

[2] Gilbert C, Tang T-C, Ott W, Dorr BA, Shaw WM, Sun GL, Lu TK, and Ellis T (2021). Living materials with programmable functionalities grown from engineered microbial co-cultures. Nat. Mater 20, 691–700. 10.1038/s41563-020-00857-5. 33432140

[3] Liu AP, Appel EA, Ashby PD, Baker BM, Franco E, Gu L, Haynes K, Joshi NS, Kloxin AM, Kouwer PHJ, (2022). The living interface between synthetic biology and biomaterial design. Nat. Mater 21, 390–397. 10.1038/s41563-022-01231-3. 35361951 PMC10265650

[4] Tang T-C, An B, Huang Y, Vasikaran S, Wang Y, Jiang X, Lu TK, and Zhong C (2020). Materials design by synthetic biology. Nat. Rev. Mater 6, 332–350. 10.1038/s41578-020-00265-w.

[5] Chen F, and Wegner SV (2022). Photoswitchable Bacterial Adhesions for the Control of Multicellular Behavior, pp. 129–148. 10.1007/978-3-030-92949-7_5.

[6] Kim H, Jin X, Glass DS, and Riedel-Kruse IH (2020). Engineering and modeling of multicellular morphologies and patterns. Curr. Opin. Genet. Dev 63, 95–102. 10.1016/j.gde.2020.05.039. 32629326

[7] Glass DS, and Riedel-Kruse IH (2018). A synthetic bacterial cell-cell adhesion toolbox for programming multicellular morphologies and patterns. Cell 174, 649–658.e16. 10.1016/j.cell.2018.06.041. 30033369

[8] Park S, Shou W, Makatura L, Matusik W, and Fu KK (2022). 3d printing of polymer composites: Materials, processes, and applications. Matter 5, 43–76. 10.1016/j.matt.2021.10.018.

[9] Timmis K, Timmis JK, Brüssow H, and Fernández LÁ (2019). Synthetic consortia of nanobody-coupled and formatted bacteria for prophylaxis and therapy interventions targeting microbiome dysbiosis-associated diseases and co-morbidities. Microb. Biotechnol 12, 58–65. 10.1111/1751-7915.13355. 30575298 PMC6302794

[10] Chen F, Zeng W, and Wegner SV (2022a). Ultrasound-activated bacteria with thermostat controls as living therapeutics. Matter 5, 2416–2419. 10.1016/j.matt.2022.05.014.

[11] Jin X, and Riedel-Kruse IH (2018). Biofilm lithography enables high-resolution cell patterning via optogenetic adhesin expression. Proc. Natl. Acad. Sci. USA 115, 3698–3703. 10.1073/pnas.1720676115. 29555779 PMC5889658

[12] Kim H, Skinner DJ, Glass DS, Hamby AE, Stuart BAR, Dunkel J, and Riedel-Kruse IH (2022). 4-bit adhesion logic enables universal multicellular interface patterning. Nature 608, 324–329. 10.1038/s41586-022-04944-2. 35948712 PMC9365691

[13] Ye X, Zhao L, Liang J, Li X, and Chen G-Q (2017). Study of the tensile properties of individual multicellular fibres generated by bacillus subtilis. Sci. Rep 7, 46052–46115. 10.1038/srep46052. 28378797 PMC5380956

[14] Salema V, and Fernández LÁ. (2017). Escherichia coli surface display for the selection of nanobodies. Microb. Biotechnol 10, 1468–1484. 10.1111/1751-7915.12819. 28772027 PMC5658595

[15] Chen B, Kang W, Sun J, Zhu R, Yu Y, Xia A, Yu M, Wang M, Han J, Chen Y, (2022b). Programmable living assembly of materials by bacterial adhesion. Nat. Chem. Biol 18, 289–294. 10.1038/s41589-021-00934-z. 34934187

[16] Rasoulinejad S, Mueller M, Nzigou Mombo B, and Wegner SV (2020). Orthogonal blue and red light controlled cell–cell adhesions enable sorting-out in multicellular structures. ACS Synth. Biol 9, 2076–2086. 10.1021/acssynbio.0c00150. 32610009 PMC7757848

[17] Chao G, Wannier TM, Gutierrez C, Borders NC, Appleton E, Chadha A, Lebar T, and Church GM (2022). helixcam: A platform for programmable cellular assembly in bacteria and human cells. Cell 185, 3551–3567.e39. 10.1016/j.cell.2022.08.012. 36055250 PMC9481732

[18] Stevens AJ, Harris AR, Gerdts J, Kim KH, Trentesaux C, Ramirez JT, McKeithan WL, Fattahi F, Klein OD, Fletcher DA, and Lim WA (2023). Programming multicellular assembly with synthetic cell adhesion molecules. Nature 614, 144–152. 10.1038/s41586-022-05622-z. 36509107 PMC9892004

[19] Chen P-Y, Chen Y-C, Chen P-P, Lin K-T, Wang W-L, Hsia K-C, and Ting S-Y (2023). A whole-cell screening platform to discover cell adhesion molecules that enable programmable bacterial cell-cell adhesion. bioRxiv, 2023–2112. 10.1101/2023.12.03.569830.

[20] Molinari S, Tesoriero RF, and Ajo-Franklin CM (2021). Bottom-up approaches to engineered living materials: Challenges and future directions. Matter 4, 3095–3120. 10.1016/j.matt.2021.08.001.

[21] Mutalik VK, Guimaraes JC, Cambray G, Mai Q-A, Christoffersen MJ, Martin L, Yu A, Lam C, Rodriguez C, Bennett G, (2013). Quantitative estimation of activity and quality for collections of functional genetic elements. Nat. Methods 10, 347–353. 10.1038/nmeth.2403. 23474467

[22] Borujeni AE, Zhang J, Doosthosseini H, Nielsen AA, and Voigt CA (2020). Genetic circuit characterization by inferring rna polymerase movement and ribosome usage. Nat. Commun 11, 1–18. 10.1038/s41467-020-18630-2. 33020480 PMC7536230

[23] McBride CD, and Del Vecchio D (2021). Predicting composition of genetic circuits with resource competition: demand and sensitivity. ACS Synth. Biol 10, 3330–3342. 10.1021/acssynbio.1c00281. 34780149

[24] Nielsen AAK, Der BS, Shin J, Vaidyanathan P, Paralanov V, Strychalski EA, Ross D, Densmore D, and Voigt CA (2016). Genetic circuit design automation. Science 352, aac7341. 10.1126/science.aac7341. 27034378

[25] Canton B, Labno A, and Endy D (2008). Refinement and standardization of synthetic biological parts and devices. Nat. Biotechnol 26, 787–793. 10.1038/nbt1413. 18612302

[26] Schaffner M, Rühs PA, Coulter F, Kilcher S, and Studart AR (2017). 3d printing of bacteria into functional complex materials. Sci. Adv 3, eaao6804. 10.1126/sciadv.aao6804. 29214219 PMC5711516

[27] Duraj-Thatte AM, Manjula-Basavanna A, Rutledge J, Xia J, Hassan S, Sourlis A, Rubio AG, Lesha A, Zenkl M, Kan A, (2021). Programmable microbial ink for 3d printing of living materials produced from genetically engineered protein nanofibers. Nat. Commun 12, 6600. 10.1038/s41467-021-26791-x. 34815411 PMC8611031

[28] Gregor T, Tank DW, Wieschaus EF, and Bialek W (2007). Probing the limits to positional information. Cell 130, 153–164. 10.1016/j.cell.2007.05.025. 17632062 PMC2253670

[29] Schneider F, Sych T, Eggeling C, and Sezgin E (2021). Influence of nanobody binding on fluorescence emission, mobility, and organization of gfp-tagged proteins. iScience 24, 101891. 10.1016/j.isci.2020.101891. 33364580 PMC7753935

[30] Salema V, Marín E, Martínez-Arteaga R, Ruano-Gallego D, Fraile S, Margolles Y, Teira X, Gutierrez C, Bodelón G, and Fernández LÁ (2013). Selection of single domain antibodies from immune libraries displayed on the surface of e. coli cells with two β-domains of opposite topologies. PLoS One 8, e75126. 10.1371/journal.pone.0075126. 24086454 PMC3781032

[31] Ellis T, Wang X, and Collins JJ (2009). Diversity-based, model-guided construction of synthetic gene networks with predicted functions. Nat. Biotechnol 27, 465–471. 10.1038/nbt.1536. 19377462 PMC2680460

[32] Da Re S, Le Quéré B, Ghigo J-M, and Beloin C (2007). Tight modulation of escherichia coli bacterial biofilm formation through controlled expression of adhesion factors. Appl. Environ. Microbiol 73, 3391–3403. 10.1128/AEM.02625-06. 17384304 PMC1907114

[33] Alon U (2019). An Introduction to Systems Biology: Design Principles of Biological Circuits

[34] Ang J, Harris E, Hussey BJ, Kil R, and McMillen DR (2013). Tuning response curves for synthetic biology. ACS Synth. Biol 2, 547–567. 10.1021/sb4000564. 23905721 PMC3805330

[35] Gardner TS, Cantor CR, and Collins JJ (2000). Construction of a genetic toggle switch in escherichia coli. Nature 403, 339–342. 10.1038/35002131. 10659857

[36] Mandelstam J (1958). Turnover of protein in growing and non-growing populations of escherichia coli. Biochem. J 69, 110–119. 10.1042/bj0690110. 13535591 PMC1196522

[37] Kalisky T, Dekel E, and Alon U (2007). Cost–benefit theory and optimal design of gene regulation functions. Phys. Biol 4, 229–245. 10.1088/1478-3975/4/4/001. 17991990

[38] Young JW, Locke JCW, Altinok A, Rosenfeld N, Bacarian T, Swain PS, Mjolsness E, and Elowitz MB (2011). Measuring single-cell gene expression dynamics in bacteria using fluorescence time-lapse microscopy. Nat. Protoc 7, 80–88. 10.1038/nprot.2011.432. 22179594 PMC4161363

[39] Maurizi MR (1992). Proteases and protein degradation in escherichia coli. Experientia 48, 178–201. 10.1007/BF01923511. 1740190

[40] Baneyx F, and Georgiou G (1990). In vivo degradation of secreted fusion proteins by the escherichia coli outer membrane protease ompt. J. Bacteriol 172, 491–494. 10.1128/jb.172.1.491-494.1990. 2403549 PMC208460

[41] Schroer DW, and St John AC (1981). Relative stability of membrane proteins in escherichia coli. J. Bacteriol 146, 476–483. 10.1128/jb.146.2.476-483.1981. 7012130 PMC216989

[42] Gottesman S (1996). Proteases and their targets in escherichia coli. Annu. Rev. Genet 30, 465–506. 10.1146/annurev.genet.30.1.465. 8982462

[43] Axelrod D, Koppel DE, Schlessinger J, Elson E, and Webb WW (1976). Mobility measurement by analysis of fluorescence photobleaching recovery kinetics. Biophys. J 16, 1055–1069. 10.1016/S0006-3495(76)85755-4. 786399 PMC1334945

[44] Spector J, Zakharov S, Lill Y, Sharma O, Cramer WA, and Ritchie K (2010). Mobility of btub and ompf in the escherichia coli outer membrane: implications for dynamic formation of a translocon complex. Biophys. J 99, 3880–3886. 10.1016/j.bpj.2010.10.029. 21156129 PMC3000481

[45] Verhoeven GS, Dogterom M, and den Blaauwen T (2013). Absence of long-range diffusion of ompa in e. coli is not caused by its peptidoglycan binding domain. BMC Microbiol 13, 66–69. 10.1186/1471-2180-13-66. 23522061 PMC3637615

[46] Leo JC, Oberhettinger P, Chaubey M, Schütz M, Kühner D, Bertsche U, Schwarz H, Götz F, Autenrieth IB, Coles M, and Linke D (2015). The intimin periplasmic domain mediates dimerisation and binding to peptidoglycan. Mol. Microbiol 95, 80–100. 10.1111/mmi.12840. 25353290

[47] Rassam P, Copeland NA, Birkholz O, Tóth C, Chavent M, Duncan AL, Cross SJ, Housden NG, Kaminska R, Seger U, (2015). Supramolecular assemblies underpin turnover of outer membrane proteins in bacteria. Nature 523, 333–336. 10.1038/nature14461. 26061769 PMC4905513

[48] Visscher K, Gross S, and Block S (1996). Construction of multiple-beam optical traps with nanometer-resolution position sensing. IEEE J. Sel. Top. Quant. Electron 2, 1066–1076. 10.1109/2944.577338.

[49] Koch MD, and Shaevitz JW (2017). Introduction to optical tweezers. Methods Mol. Biol 1486, 3–24. 10.1007/978-1-4939-6421-5_1. 27844423

[50] Markowska A, Markowski AR, and Jarocka-Karpowicz I (2021). The importance of 6-aminohexanoic acid as a hydrophobic, flexible structural element. Int. J. Mol. Sci 22, 12122. 10.3390/ijms222212122. 34830000 PMC8618066

[51] El-Kirat-Chatel S, Mil-Homens D, Beaussart A, Fialho AM, and Dufrêne YF (2013). Single-molecule atomic force microscopy unravels the binding mechanism of a b urkholderia cenocepacia trimeric autotransporter adhesin. Mol. Microbiol 89, 649–659. 10.1111/mmi.12301. 23796134

[52] Klamecka K, Severin PM, Milles LF, Gaub HE, and Leonhardt H (2015). Energy profile of nanobody–gfp complex under force. Phys. Biol 12, 056009. 10.1088/1478-3975/12/5/056009. 26356046

[53] Viji Babu PK, Mirastschijski U, Belge G, and Radmacher M (2021). Homophilic and heterophilic cadherin bond rupture forces in homo-or hetero-cellular systems measured by afm-based single-cell force spectroscopy. Eur. Biophys. J 50, 543–559. 10.1007/s00249-021-01536-2. 33880610 PMC8190030

[54] Wang X, Chen Q, Sun Z, Wang Y, Su B, Zhang C, Cao H, and Liu X (2020). Nanobody affinity improvement: Directed evolution of the anti-ochratoxin a single domain antibody. Int. J. Biol. Macromol 151, 312–321. 10.1016/j.ijbiomac.2020.02.180. 32084462

[55] Zhang Z, Wang Y, Ding Y, and Hattori M (2020). Structure-based engineering of anti-gfp nanobody tandems as ultra-high-affinity reagents for purification. Sci. Rep 10, 6239. 10.1038/s41598-020-62606-7. 32277083 PMC7148334

[56] Zakeri B, Fierer JO, Celik E, Chittock EC, Schwarz-Linek U, Moy VT, and Howarth M (2012). Peptide tag forming a rapid covalent bond to a protein, through engineering a bacterial adhesin. Proc. Natl. Acad. Sci. USA 109, E690–E697. 10.1073/pnas.1115485109. 22366317 PMC3311370

[57] Maier B (2021). How physical interactions shape bacterial biofilms. Annu. Rev. Biophys 50, 401–417. 10.1146/annurev-biophys-062920-063646. 33637007

[58] Williams SR, and Philipse AP (2003). Random packings of spheres and spherocylinders simulated by mechanical contraction. Phys. Rev 67, 051301. 10.1103/PhysRevE.67.051301. 12786140

[59] Kittel C, and McEuen P (2018). Introduction to Solid State Physics

[60] Lutz R, and Bujard H (1997). Independent and tight regulation of transcriptional units in escherichia coli via the lacr/o, the tetr/o and arac/i1-i2 regulatory elements. Nucleic Acids Res 25, 1203–1210. 10.1093/nar/25.6.1203. 9092630 PMC146584

[61] Liu Z, Zhang Z, and Ritchie RO (2020). Structural orientation and anisotropy in biological materials: functional designs and mechanics. Adv. Funct. Mater 30, 1908121. 10.1002/adfm.201908121.

[62] Copenhagen K, Alert R, Wingreen NS, and Shaevitz JW (2021). Topological defects promote layer formation in myxococcus xanthus colonies. Nat. Phys 17, 211–215. 10.1038/s41567-020-01056-4. PMC12395370 40893480

[63] Fridy PC, Li Y, Keegan S, Thompson MK, Nudelman I, Scheid JF, Oeffinger M, Nussenzweig MC, Fenyö D, Chait BT, and Rout MP (2014). A robust pipeline for rapid production of versatile nanobody repertoires. Nat. Methods 11, 1253–1260. 10.1038/nmeth.3170. 25362362 PMC4272012

[64] Tay PKR, Nguyen PQ, and Joshi NS (2017). A synthetic circuit for mercury bioremediation using self-assembling functional amyloids. ACS Synth. Biol 6, 1841–1850. 10.1021/acssynbio.7b00137. 28737385

[65] An B, Wang Y, Jiang X, Ma C, Mimee M, Moser F, Li K, Wang X, Tang T-C, Huang Y, (2020). Programming living glue systems to perform autonomous mechanical repairs. Matter 3, 2080–2092. 10.1016/j.matt.2020.09.006.

[66] López Barreiro D, Yeo J, Tarakanova A, Martin-Martinez FJ, and Buehler MJ (2019). Multiscale modeling of silk and silk-based biomaterials—a review. Macromol. Biosci 19, 1800253. 10.1002/mabi.201800253. 30375164

[67] Bounoua S, Lemaire E, Férec J, Ausias G, and Kuzhir P (2016). Shear-thinning in concentrated rigid fiber suspensions: Aggregation induced by adhesive interactions. J. Rheol 60, 1279–1300. 10.1122/1.4965431.

[68] Shahmohammadi A, and Bonnecaze RT (2021). Linear viscoelastic properties of adhesive soft particle glasses. J. Rheol 65, 463–475. 10.1122/8.0000239.

[69] Sankaran S, Becker J, Wittmann C, and Del Campo A (2019). Optoregulated drug release from an engineered living material: self-replenishing drug depots for long-term, light-regulated delivery. Small 15, 1804717. 10.1002/smll.201804717. 30589209

[70] Donderwinkel I, van Hest JCM, and Cameron NR (2017). Bio-inks for 3d bioprinting: recent advances and future prospects. Polym. Chem 8, 4451–4471. 10.1039/C7PY00826K.

