# Supplementary material for: Biophysical characterization of synthetic adhesins for predicting and tuning engineered living material properties
Source: Matter. Author manuscript; Available in PMC 2024 Aug 20. (PMC11335339; doi:10.1016/j.matt.2024.03.019)
Supplement: MMC1 [file NIHMS1985003-supplement-MMC1.pdf]

Matter, Volume 7

Supplemental information

Biophysical characterization of synthetic adhesins for predicting and tuning engineered living material properties

Stefana A. Costan, Paul M. Ryan, Honesty Kim, Charles W. Wolgemuth, and Ingmar H. Riedel-Kruse

Matter, Volume *7*

Supplemental information

Biophysical characterization of synthetic adhesins for predicting and tuning engineered living material properties

Stefana A. Costan, Paul M. Ryan, Honesty Kim, Charles W. Wolgemuth, and Ingmar H. Riedel-Kruse

Supplemental Note 1: Number of adhesins and spatial surface distribution

Model for determining the number of adhesins:

The following protocol is adapted from[1].

The fluorescence intensity value per pixel is measured for the parameters shown in table S1 (compare also Figure 2B):

Parameter	Description
I_{GFP}	fluorescence intensity of anti-GFP cells labeled with GFP and bathed in a solution of GFP of known concentration
I^{β}_{GFP}	fluorescence intensity of the background of the anti-GFP cells labeled with GFP and bathed in a solution of GFP of known concentration
I_{WT}	fluorescence intensity of the wild-type cells bathed in a solution of GFP of known concentration
I^{β}_{WT}	fluorescence intensity of the background of the WT cells bathed in a solution of GFP of known concentration
I_{AF}	fluorescence of the background of the control cells (no GFP added)
I'_{GFP}	corrected fluorescence intensity of anti-GFP cells labeled with GFP and bathed in a solution of GFP
I'^{β}_{GFP}	corrected fluorescence intensity of the background of the anti-GFP cells labeled with GFP and bathed in a solution of GFP
$I'_{GFP,eff}$	effective fluorescence intensity of the anti-GFP cells labeled with GFP and bathed in a solution of GFP
C_{GFP}	concentration of the GFP solution

Table S1. Parameters of the model

The following corrections are made: The true intensity contributed by the cell, where the GFP is bound to its surface is calculated by correcting for the background:

$$I'_{GFP} = I_{GFP} - \frac{I_{WT}}{I^{\beta}_{WT}} I^{\beta}_{GFP} \tag{S1}$$

The correction for background autofluorescence:

$$I'^{\beta}_{GFP} = I^{\beta}_{GFP} - I_{AF} \tag{S2}$$

A voxel is determined by using the pixel area and the confocal axial PSF (Point Spread Function) (pinhole ≥ 1 AU)[2]:

$$V_{vox} = A_{pix} \cdot FWHM_{ill,axial} \tag{S3}$$

$$FWHM_{ill,axial} = \frac{0.88 \cdot \lambda_{exc}}{n - \sqrt{n^2 - NA^2}} \tag{S4}$$

where λ_{exc} is the wavelength of illuminating light, n is the refractive index of the medium between the cover slip and the objective front lens element, and NA equals the objective numerical aperture.

Then, the effective fluorescence intensity of the GFP cell, (labeled with GFP and bathed in a GFP solution) is:

$$I'_{GFP,eff} = I'_{GFP} \cdot \frac{N_{pix,slice}}{1/2 A_{cell,pix}} \tag{S5}$$

where $N_{pix,slice}$ is the number of pixels in the confocal slice that belong to the cell and $A_{cell,pix}$ is the area of the cell in

units of pixel. This correction is necessary because the axial PSF is about equal to the radius of the cell so the intensity from top and bottom are not considered simultaneously.

The GFP solution is of known concentration, 365 nM. According to Avogadro's number, 1 nM corresponds to 0.6 molecules/μm^3, therefore in the GFP solution there are 220 molecules/μm^3. Here "molecules" denotes the number of GFP molecules which later will be the equivalent to the number of adhesins on the surface of the cell (assuming 1 GFP represents 1 labeled adhesin).

The surface of the *E. coli* cells is computed using the surface area of a capsule:

$$A_{cell} = 2\pi rl, \quad \text{(S6)}$$

where l is the total length and r is the radius of the cell.

Assuming that all the fluorescent adhesins in the voxel are located on the surface of the bacterium, the number of adhesins per cell can finally be determined by:

$$\frac{N_{adh}}{cell} = \frac{I'_{GFP,eff}}{I'^{\beta}_{GFP}} \cdot 0.6 \cdot C_{GFP} \cdot V_{vox} \cdot \frac{1}{A_{pix}} \cdot \frac{A_{cell}}{cell} \quad \text{(S7)}$$

A sample calculation using Table S1 parameters is presented in Table S2 for 300 ng/mL aTc induction level.

**Parameter**	**Value in A.U (mean ± sem)**
I_{GFP}	38.20 ± 0.62
I^{β}_{GFP}	1.08 ± 0.08
I_{WT}	1.63 ± 0.05
I^{β}_{WT}	1.04 ± 0.02
I_{AF}	0.07 ± 0.01

Table S2. Fluorescence intensity values for the parameters of the model. n = 20 cells

Measured dimensions of *E. coli* cells are as follows:

**Parameter**	**Description**	**Unit**	**Value (mean ± sem)**
r	Cell radius	μm	0.4 ± 0.1
l	Total cell length	μm	1.80 ± 0.25
L	Cylindrical part length	μm	1.00 ± 0.15
A_{cell}	Surface area of the cell	μm^2	4.48 ± 0.69
V_{cell}	Volume of the cell	μm^3	0.96 ± 0.14

Table S3. Measured dimensions for the imaged cells. n = 20 cells

For the imaging setup the image size is predefined to be 16.9 x 16.9 microns or 188 x 188 pixels. This was done using the Optimal function in the microscope software that sets the image resolution to an optimal value corresponding to the optical magnification (objective) the zoom and the emission range detected. Therefore the area of a pixel is 0.0081 μm^2. The, $FWHM_{ill,axial}$ for the confocal axial point spread function (pinhole > 1 AU) is 0.45 μm given the 488 nm excitation wavelength, refractive index for the immersion oil of 1.51 and the NA for the 63x objective of 1.4.

The standard error associated with uncertainty propagation is determined through a Monte Carlo simulation. Values and errors are presented in the "Error analysis" subsection below.

The numbers of adhesins for the 300 ng/mL induction level are then quantified to be:

$$N_{adh}(300) = 15,400 \pm 4,100 \text{ adhesins/cell (mean} \pm \text{sem)}$$

Estimating the number of adhesins per cell based based on pixel variations

In Figure 2D the average fluorescence per pixel is measured along the circumference of the cell for 10 cells and a normal distribution is fitted with the mean at $N_m = 41$ A.U./pixel, the variance $N_v = 16$ and a standard deviation of $N_s = 4$ A.U./pixel.

Given the measured dimensions of the cell, a surface area of the cell of $4.5 \pm 0.7\ \mu m^2$ is calculated. As the area of the pixel is 0.0081 μm^2, the number of pixels on the surface of a cell is:

$$N_{pix} = 555.6 \pm 0.2 \text{ (mean } \pm \text{ sem)}$$

Assuming that each of the N_{adh} adhesins in a cell is randomly assigned to any of the N_{pix} pixels, the number of adhesins for 300 ng/mL induction level is expected to be:

$$N_{adh}(300) = (N_m/\sqrt{N_v})^2 * N_{pix} = (N_m/N_s)^2 * N_{pix} \tag{S8}$$

$$N_{adh}(300) = 57,800 \text{ adhesins/cell}$$

with a value of adhesins per pixel of $N_{adh,pix}$ = 105. This statement is confirmed with a corresponding Monte Carlo simulation based on the previously measured number of adhesins by direct calibration. The number of adhesins per pixels is determined to be 27.6 ± 5.1 (mean ± std).

The resulting value of $N_{adh}(300)$ = 57,800 adhesins per cell and $N_{adh,pix}$ = 105 based on pixel variation, is in same order of magnitude but higher than what was measured by direct calibration earlier. This discrepancy in the number of adhesins per pixel determined through pixel variation compared to direct calibration can likely be understood as the cell outline where this measurement was carried out represents a top down projection of the cell edge containing many more adhesins.

Error analysis

Random error:

To determine the random error, the experiment is repeated twice, on different days, and each repeat consists of 10 cells. The results between the two days of experiments are compared via the Student's t-test, and no statistical significant difference is observed (Student's t-test, p = 0.8). Therefore, all the data (20 cells total, across 2 different days) is combined to determine the parameters in the model. Because each parameter in the model comes with its own error (standard error of the mean, see Tables S2 and S3), a Monte-Carlo simulation is run to determine the propagation of uncertainty. For this simulation, normal data (100 points) is generated for each parameter using the mean and sem (Table S4) and then the final equation to determine the number of adhesins is computed[3].

**aTc (ng/mL)**	**N_{adh}/cell (mean± sem)**
300	15,400 ± 4,100
100	7,300 ± 1,800
30	170 ± 70

Table S4. Number of adhesins per cell calculated from the fluorescence data

Systematic error:

Systematic errors are associated with the equipment used[3]. The errors that affect the quantification of the number of adhesins are:

- Pipetting GFP into the solution: For the Gilson pipette: 0.8% error is presented in the manual. This is only applicable for the comparison with the solution of a known concentration of GFP (not relevant for the fluorescence intensity of the cells because GFP is always added in excess and the unbound protein is washed). Therefore, the error will be taken into consideration for the fluorescence intensity: I^{β}_{GFP} and I^{β}_{WT}. For a concentration of 365 nM the error would be ± 3 nM.
- Pipetting inducer into the solution: That means that the fluorescence intensity for the cells observed through imaging has a systematic error of 0.8%.

- Recombinant GFP stock solution concentration presented in the Certificate of Analysis by Clontech: 10%. Again, this is only applicable for the comparison with the solution of a known concentration of GFP (not relevant for the fluorescence intensity of the cells because GFP is always added in excess and the unbound protein is washed).

- The light in the SCM is highly coherent (a single-mode laser is used for illumination), so the image does not suffer from the systematic errors associated with partially coherent illumination[4]. However there are systematic error associated with the diameter of the pinhole of 0.1 %[4]. Because of the model and the corrections we applied, these errors balance each other out so they become negligible.

  Associating each parameter in the model with its corresponding systematic error and generating normal data using the mean and this error, a Monte Carlo simulation is run to determine the adhesin number systematic error for 100 ng/mL aTc:

$$N_{adh}(100) = 7,300 \pm 1,500 \text{ molecules/cell} \tag{S9}$$

Supplemental Note 2: Kinetics of adhesin turnover

Model for determining the kinetics of adhesin turnover:

The relationship between measured fluorescence intensity I(t) and the number adhesins per cell p(t) is always given by the calibration factor f, i.e.,:

$$I(t) = f \cdot p(t) \tag{S10}$$

The kinetics of p(t) over time t is given by the maximum protein production rate a_{max} and degradation rate b and depends on C[5]:

$$\dot{p}(t) = a_{max} \frac{C^n}{C^n + C_{1/2}^n} - b \cdot p(t) \tag{S11}$$

with n being the Hill coefficient and $C_{1/2}$ the inducer concentration for half-maximal expression.

This also motivates the definition of an effective concentration production rate a(C) for any given induction level C:

$$a(C) = a_{max} \frac{C^n}{C^n + C_{1/2}^n} \tag{S12}$$

The ratio between production and degradation rates represents the number of adhesins $\tilde{p}$ (or termed $N_{adh}(C)$) at steady state expression for a given induction level:

$$N_{adh}(C) = \tilde{p} = \frac{a(C)}{b} = \frac{a_{max}}{b} \frac{C^n}{C^n + C_{1/2}^n} \tag{S13}$$

Generally, b itself is an effective rate due to multiple contributions: $b = b_{eff} = b_{deg} + b_{dil}$, were b_{deg} is the specific degradation rate due to physical protein destruction and b_{dil} is the protein dilution rate due to cell division, and where $b_{dil} >> b_{deg}$ while cells are dividing, and $b_{dil} = 0$ while cells are not dividing under starving conditions[5].

Equation (S11) has then the following solutions relevant for the type of experiment that were carried out, where either b_{deg} or b_{dil} does not play any significant role:

CASE A: Cells dividing

Initially there are no adhesins (p(t=0)=0), and at t = 0 adhesion expression is induced and cells are growing and dividing. Hence $b_{eff} \approx b_{dil}$. The adhesin concentration then changes over time according to:

$$p(t) = \frac{a_{max}}{b_{eff}} \frac{C^n}{C^n + C_{1/2}^n} \cdot (1 - e^{-t \cdot b_{eff}}) \tag{S14}$$

This then leads to the steady state value of p of:

$$p(t \to \infty) = \tilde{p} = \frac{a_{max}}{b_{eff}} \cdot \frac{C^n}{C^n + C_{1/2}^n} \tag{S15}$$

Using this conversion factor, f, protein expression can be converted into fluorescence dynamics.

$$I(t) = f \cdot \frac{a_{max}}{b_{eff}} \cdot \frac{C^n}{C^n + C_{1/2}^n} \cdot (1 - e^{-t \cdot b_{eff}}) = f \cdot \frac{a(C)}{b_{eff}} \cdot (1 - e^{-t \cdot b_{eff}}) = f \cdot N_{adh}(C) \cdot (1 - e^{-t \cdot b_{eff}}) \tag{S16}$$

By fitting the fluorescence intensity over time for cells that were transferred from medium without inducer to medium with inducer (Figure 3A,B) and knowing f, $C_{1/2}$, and n from the "Number of adhesins and spatial surface distribution", the maximum protein production rate, a_{max} , and the effective decay rate, b_{eff}, are determined:

$$a_{max} = 9,900 \pm 2,100 \text{ adhesins/cell/h}$$

For an induction level of 100 ng/mL aTc, the production rate is $a(100) = 5,600 \pm 1,200$ adhesins/cell/h.

$$b_{eff} = 0.78 \pm 0.12 \text{ 1/h}$$

Here $b_{eff} \approx b_{dil}$, but in order to verify this, the dilution rate, b_{dil} was also determined from OD600 measurements of the growing culture (Figure S1):

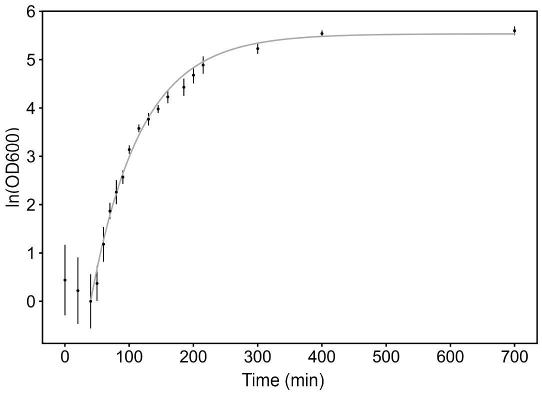

Figure S1. Cell growth monitored through the optical density values at 600 nm.
Normalized data as a function of time was fitted with an exponential equation

$$b_{dil} = 0.82 \pm 0.3\ h^{-1}$$

Therefore the effective decay rate during growth and the dilution rate are not significantly different (Student's t-test, p = 0.6), which is consistent with previous studies on other proteins[6–8].

CASE B: Cells starving

Cells have been growing and dividing and expressing adhesins according to case A and have reached this steady state level $\tilde{p}$. Because the adhesin degradation rate due to destruction cannot be determined from growing conditions since dilution rate is the predominant process, the cells are transferred at $t = 0$ to conditions where they do not divide anymore (starving conditions), and adhesin production rate is shut down (inducer is removed) (Figure 3C,D). Hence $b_{eff} \approx b_{deg}$. This then leads to the adhesin concentration to change over time according to:

$$I(t) = f \cdot \frac{a_{max}}{b_{eff}} \cdot \frac{C^n}{C^n + C_{1/2}^n} \cdot e^{-t \cdot b_{eff}} \tag{S17}$$

The specific degradation of adhesins can then be determined by fitting the exponential decay function (Figure 3E):

$$b_{deg} = 0.07 \pm 0.01\ h^{-1} \tag{S18}$$

The degradation rate of the adhesin is also often alternatively stated as any of three three equivalent parameters:

$$t_{1/2} = \frac{\ln(2)}{b_{deg}} = \tau \ln(2) \tag{S19}$$

where τ is the mean lifetime of the adhesins and $t_{1/2}$ is the half-life of the adhesins.

In one of the control experiments, a secondary agarose pads is imaged under the same condition as before but at a lower frame rate (pink) than the experimental points (black) which led to the conclusion that photofading of the GFP molecules due to imaging is negligible (Figure S2A).

In an additional control experiment, the cells are kept in liquid PBS and at various time points, samples are collected, labeled and imaged (Figure S2B). This control experiment leads to a lower degradation rate of $0.05 \pm 0.01\ h^{-1}$ (in contrast to the previously determined value of $0.07 \pm 0.01\ h^{-1}$). This indicates, that there is a possible degradation of the GFP molecule on the agarose pads or unbinding from the Nb, without the possibility of rebinding (which would happen in liquid). This result (Figure S2B) is more reliable and suggestive of the underlying molecular dynamics. The degradation rate measured from labeled cells that always stayed on agarose pads (Figure S2A) is likely overestimated by a factor of about 1.4, and therefore, the degradation rate is $0.05 \pm 0.01\ h^{-1}$ - as stated in the main paper.

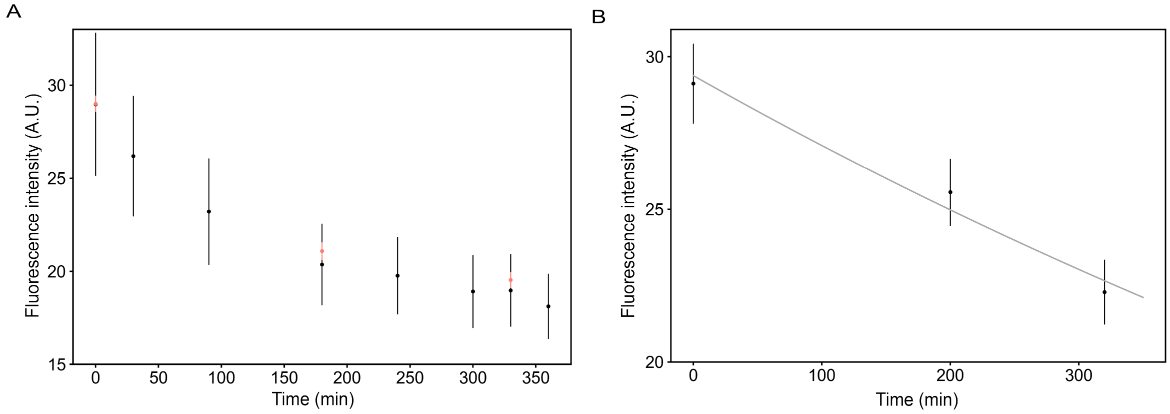

Figure S2. Adhesin degradation controls
(A) Photofading control where cells on a secondary agarose pads were imaged at a much lower frame rate (pink) compared to the experiment (black).
(B) Experimental control where cells are kept in liquid PBS, labeled and then imaged.

Error analysis
Random error:
The random error was determined similarly to the previous section to be:

$$a(100) = 5{,}600 \pm 1{,}200 \text{ adhesins/cell/h}$$

$$b_{deg} = 0.05 \pm 0.01 \text{ 1/h}$$

$$b_{dil} = 0.82 \pm 0.3 \text{ 1/h}$$

Systematic error:

- Pipetting inducer into the solution: For the Gilson pipette: 0.8 % error presented in the manual. That means that the fluorescence intensity observed through imaging has a systematic error of 0.8 %.
- Recombinant GFP stock solution concentration presented in the Certificate of Analysis of the product: 10%. It is not relevant for the fluorescence intensity of the cells because GFP is always added in excess and the unbound protein is washed.
- There are systematic error associated with the diameter of the pinhole of 0.1 %[4] which apply to the overall fluorescence intensity observed over time.
- For the Nanodrop 1000: 0.5% for 2 μL of sample. This is relevant for calculating the dilution rate.

The systematic error is determined through a Monte-Carlo simulation:

$$a(100) = 5{,}600 \pm 900 \text{ adhesins/cell/h}$$

$$b_{deg} = 0.05 \pm 0.01 \text{ 1/h}$$

$$b_{dil} = 0.82 \pm 0.1 \text{ 1/h}$$

Supplemental Note 3: Diffusion coefficient

FRAP considerations

These are a few considerations to keep in mind when setting up the FRAP experiment[9,10]. In order to monitor rapid recovery kinetics and to minimize photobleaching during acquisition, a faster scan speed can be used to decrease the pixel dwell time but there is a limit to this imposed by the equipment. To achieve a fast scan speed, in the order of miliseconds, the pixel number can be lowered by decreasing the pixel resolution (e.g. 32 x 32 instead of 512 x 512). For fluorophores that are susceptible to photobleaching at low laser intensities, opening the pinhole leads to a brighter signal with less laser power. Furthermore, frame or line averaging should be avoided to reduce undesired photobleaching in the imaging mode. Regarding the acquisition time and the frequency, the ideal postbleach acquisition duration is 5-10 times higher than the halftime[9]. In order to determine this parameter, initial experiments should be conducted until no noticeable further increase in fluorescence intensity is detected. The acquisition frequency should be adjusted to resolve the dynamic range of the recovery with good temporal resolution (rule of thumb: at least 20 data points during the time required for the half of the recovery)[11].

A circular bleach region inside the cell can be drawn using the Zeiss Zen Black software ROI selection tool. The correct size for the bleaching ROI needs to be determined on a case-by-case basis. If the bleach region is too small, fluorescence recovery may occur so quickly that it will become difficult to analyze the FRAP curve, or the FRAP curve may be very noisy. On the other hand, the ROI should not be so large that a significant fraction of the total fluorophores is lost due to the bleaching event, as this will artificially lower the mobile fraction[12]. In order to keep the ratio between the bleached spot and the size of the cell appropriate, the cells can be elongated with the antibiotic cephalexin, which blocks cell division but allows further elongation[13]. The mechanism of cephalexin affecting bacterial cells was previously elucidated: it binds with high affinity to PBP3, which only interacts with PBP2 (part of the protein complex responsible for elongation) during division at mid-cell[14]. Taking this into consideration, there should not be any differences between the structure of the cell wall in filamentous cells and that of normal length cells. Finally, FRAP results should not be over–interpreted by applying complicated FRAP analysis protocols for which underlying assumptions do not reasonably approximate experimental setups. Application of simple exponential fit of the recovery curves with few free parameters is often preferable in practice over sophisticated FRAP fitting models trying to mimic the underlying reaction–diffusion complexity in detail[10].

Model for determining the diffusion coefficient:

The fluorescence intensity of a circular region of interest for both a bleached cell and an unbleached cell in the same frame pre and post bleaching is measured using ImageJ.

First the fluorescence intensity of the bleached area over time is corrected for photofading due to imaging using a reference cell in the same frame that was not bleached, using a double normalization[15]:

$$\mathrm{Corr}(t) = \frac{\mathrm{Ref}_{\mathrm{pre-bleach}}}{\mathrm{Ref}(t)} \cdot \frac{\mathrm{FRAP}(t)}{\mathrm{FRAP}_{\mathrm{pre-bleach}}} \tag{S20}$$

The recovery was fitted with the following equation to find the half–time of recovery[10,16]:

$$\mathrm{Corr}(t) = I_0 + I_1 \cdot (1 - e^{-\tau_D \cdot t}) \tag{S21}$$

where I_0 is the normalised intensity just after bleach, I_1 is the dynamic range of recovery and τ_D is the fluorescence recovery time.

The mobile fraction, M_f is determined by[10]:

$$M_f = \frac{I_1}{1 - I_0} \tag{S22}$$

Since the bleaching profile is Gaussian, the diffusion coefficient can then be estimated using the following equation, derived from[9]:

$$D = \gamma \cdot \frac{w^2}{4t_{1/2}} \tag{S23}$$

where γ is a correction factor which is 0.88 for circular beams, w the radius of the bleaching spot and $t_{1/2}$ is the half–time of recovery and is determined by:

$$t_{1/2} = \frac{-\ln(0.5)}{\tau_D} \tag{S24}$$

The first control consists of acquiring a z stack of a bleached cell with the observation that the bleaching goes all the way through the cell (data not shown). In addition, the growth of a bleached cell on agarose pads is monitored to make sure the cells are still healthy and diffusion is occuring under normal conditions (data not shown). When fluorescence is bleached from a whole cell and the imaging was performed under the same conditions as above, there is no observable back–conversion in the first 4 seconds (Figure S3).

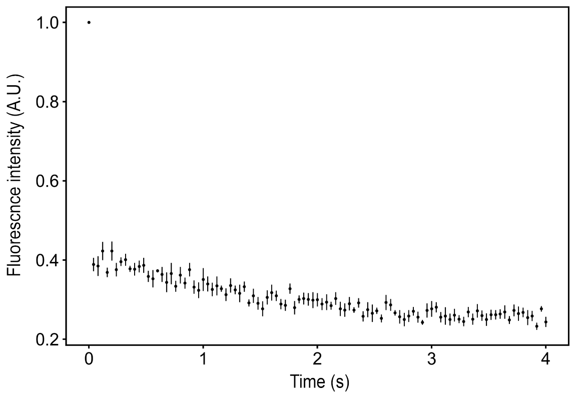

Figure S3. Fluorescence recovery monitored after bleaching the entire cell

In order to test the validity of the model, a control experiment is run with cells membrane stained with a membrane dye, SynaptoGreen. As stated in the product information sheet, in the presence of cells or tissue preparations, the dyes partition between the aqueous phase, where the dyes are virtually non–fluorescent, and the outer leaflet of the cell surface membranes, where the dyes insert the lipophilic end into the membranes and become intensely fluorescent.

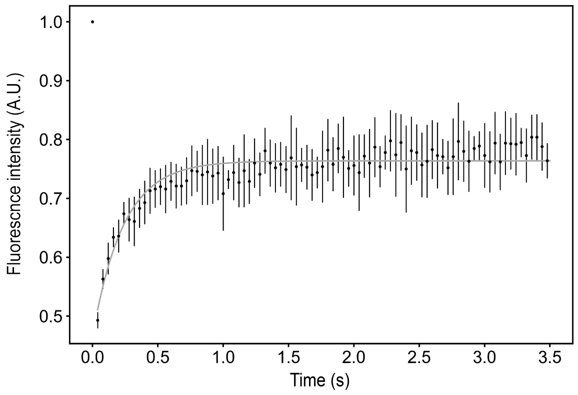

Figure S4. The membrane lipids diffusion coefficient determined through FRAP

The value for the diffusion coefficient for lipids using the model above is 0.45 ± 0.05 $\mu m^2/s$, which is consistent with literature values[17].

These results were confirmed by using an automatic software for FRAP analysis, PyFRAP[18], which led to similar results (Student's t-test, p = 0.08).

Error analysis

Random error:

The random error was determined similarly to the previous section:

$$D = 0.36 \pm 0.06\ \mu m^2/s$$

Systematic error:

- Recombinant GFP stock solution concentration presented in the Certificate of Analysis by Clontech: 10%. This is not relevant in this case as GFP is always added in excess and the unbound protein is washed.
- The light in the SCM is highly coherent (a single-mode laser is used for illumination), so the image does not suffer from the systematic errors associated with partially coherent illumination[4]. However there are systematic errors associated with the diameter of the pinhole of 0.1 %[4] which will then reflect in the value for τ from the recovery curve fit.
- Error associated with bleaching area that is attributed to the Gaussian profile of the laser of 10 %.

$$D = 0.36 \pm 0.05\ \mu m^2/s$$

Supplemental Note 4: Adhesin binding force

Determining the site of actual bond-breakage:

Bead and cell are connected to each other through a set of linkers, i.e., Ag is connected to the bead via Streptavidin and Biotin, and Nb is linked to the autotransporter inside the membrane (Figure S5). Hence when the bead is moved away from the cell with the optical trap, there are multiple points (linking elements) at a molecular level where in principle the actual breaking could be happening - besides the intended Ag-Nb bond. For each individual linking element the typical bond breaking forces are known (Figure S5):

- The connection between the polystyrene bead and Streptavidin (Figure S5 #1), as well as the one between Biotin and the antigen (Figure S5 #3) is mediated through covalent coupling. At the given force-loading rates of 10 nN/s (used in the reference), the typical forces required to break covalent bonds are around 4,000 pN[19].
- The connection between Streptavidin and Biotin (Figure S5 #2) is one of the strongest non-covalent interactions and the rupture force is dependent on the loading rate. At a loading rate between 6 and 100 pN/s (values used in the experiments) the most likely rupture force of a single Streptavidin-Biotin bond is between 30 and 60 pN[20].
- The connection between the Nb and the bacterial cell is through the autotransporter inside the membrane (Figure S5 #5). The force required to rip out an outer membrane protein from the membrane is above 300 pN, as forces below might only generate protein unfolding[21].
- There are also bond breaking force values reported for other Ag-Nb pairs than the one that was used in ourexperiments (Figure S5 #4). For example for the GFP-Nb complex in vitro a force between 28 and 56 pN was reported[22].

Based on the data in the main paper, the measured force of ~ 16pN is much lower than all the other forces involved, hence it can be safely assumed that this is the bond breaking force between the Nb and Ag. If the Nb were to be ripped out of the membrane, the Nb-Ag bond then also would be destroyed permanently - in contrast to if Nb is pulled off Ag where consequently both might be available again for future binding. Importantly, it would be largely fine for future applications if proteins were destroyed and thereby become unavailable for future binding, as the primary interest is to determine the force it takes to break two cells apart, and so it is of secondary importance what exactly breaks. It would of course make a difference whether the adhesins are still intact after a cell broke away (e.g., the Nb-Ag bond is broken) versus one or both of the adhesins being destroyed (e.g., the whole adhesin being ripped out of the membrane, or the protein ripped apart), as this could decrease the probability that these cells can again bind to each other or other cells. We investigated this through our optical trapping experiments with repeated binding/unbinding events on two different cells. We then attributed the measured force to 0, 1 or 2 bonds formed between the cell and the bead. For the first cell, we used a contact time with the bead of 1 s and recorded 26 pulling events. We compared the first 10 events (1.1 ± 0.2 bonds formed) with the last 10 events (0.5 ± 0.2 bonds formed), which indicates that there is a mild (below statistical significance, Student's t-test, $p = 0.045$) decrease in the number of bonds that are formed after several binding/unbinding events. Similarly, we tested a contact time of 2 s on a second cell and recorded 35 pulling events. The first 10 events revealed 0.6 ± 0.2 bonds formed, a similar value (not statistically significant, Student's t-test, $p = 0.4$) with 0.4 ± 0.2 bonds formed for the last 10 events (note also that the laser of the optical trap could damage some of the adhesin proteins over longer times.) We therefore conclude that the ability of two cells to bind is not (or only weakly) affected by repeatedly binding to and unbinding from each other. This would be consistent with individual adhesin molecules not being degraded during the binding-unbinding, or - given that only so few adhesins are involved in the binding between two cells (order of one) - it does not matter for many practical purposes of whether these adhesins get degraded or not.

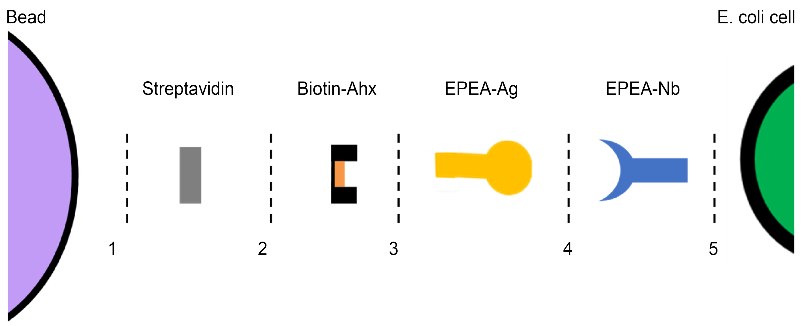

Figure S5. Bead and cell are bound to each other through a series of intermediate connections (linking elements) Each connection has a typical bond breaking force (see text for values and references); importantly: The measured force of about 16 pN with the optical trap is significantly smaller than all the known ones, therefore these events can be clearly associated with the Ag-Nb connection.

Determining the contact area between the bead and an E.coli cell

The maximum number of bonds that can be formed between a bead and an *E. coli* cell induced at different levels can be estimated as follows:

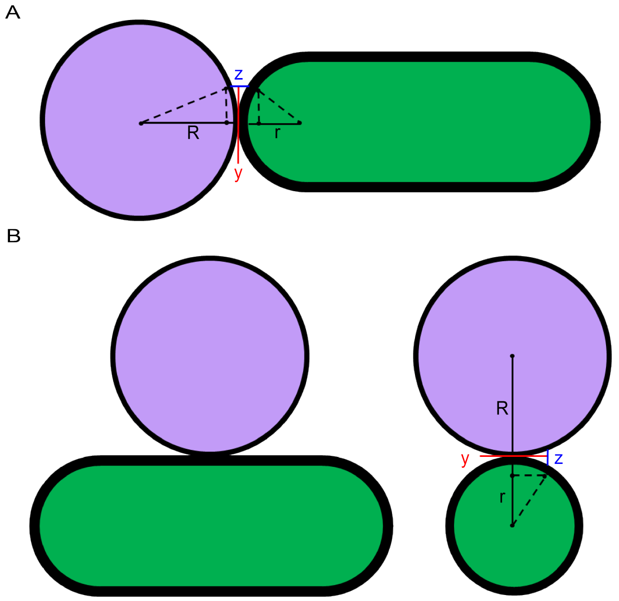

Figure S6. The possible contacts between the polystyrene bead coated with Ag and the immobilized *E. coli* cells expressing Nb
(A) Lateral contact.
(B) Top contact; see text for definitions.

The EPEA-Nb on the surface of the E.coli cell has a length of $\approx$ 125 amino acids (4 nm in length according to[23]) and also presents a spacer of 6 amino acids which in total translates to 4.2 nm length. The EPEA-Ag (small peptide, 4 amino acids, EPEA, + Ahx linker) is conjugated to biotin which binds to the Streptadivin coated beads[24,25]. The Ag-linker length is estimated to be a 1/25 fraction of the nanobody length which would be equal to 0.2 nm. The length of the Streptavidin bound to Biotin is 2.5 $\pm$ 0.1 nm[26]. Together the Ag-Nb pair including linkers to bead and cell has a total length of $z \sim 7$ nm (Figure S6A).

Based on previous measurements (Supplemental Note 1) the length of a cell is l=1.80 $\pm$ 0.25 μm and the cell radius is r= 0.40 μm. Then, considering the radius of the bead of R = 0.85 μm, the connection area available for synthetic adhesion

binding between the bacterial cell and the bead is $A = 14 \cdot 10^{-3}$ μm^2. Each cell induced at 30 ng/mL Atc expresses approximately 170 ± 70 adhesins in total over the whole surface (Supplemental Note 1). Based on these considerations, the number of bonds that can form between the bead and the cell is estimated to be between 0 and 1 when the bead touches the tip of the cell (Figure S6A). For a bead touching the cell from the top, (Figure S6B), this contact area is slightly larger ($17 \cdot 10^{-3}$ μm^2), but essentially leads to a similar conclusion.

Fitting the bond breaking force data to alternative models

A concentration of 30 ng/mL would predominantly lead to single bonds forming. The force distributions corresponding to this concentration is fitted to a sum of two Gaussian distributions centered on multiples of an unknown force value F_b.

$$G(F_b) = A_1 \cdot e^{\frac{-(F_b-\mu)^2}{2 \cdot \sigma_1{}^2}} + A_2 \cdot e^{\frac{-(F_b-2*\mu)^2}{2 \cdot \sigma_2{}^2}} \tag{S25}$$

From the fit (Figure 5D), the first distribution is centered at 16.1±0.4 pN (error from the fit) with an amplitude of 11.2±0.9 counts and a standard deviation of 4.2 ± 0.4 pN, and a second distribution centered at 32.2 ± 0.9 pN, with an amplitude of 1.7 ± 1.2 counts and a standard deviation of 2.5 ± 2.1 pN.

The possibility of forces being clustered around 10, 20 and 30 pN is excluded by similarly assuming a sum of three Gaussian functions and observing a very poor fit and unreasonable parameters (Figure S7A): first distribution centered at 8.0 ± 0.5 pN with an amplitude of $1.6 \cdot 10^{-7} \pm 2.1$ counts and a standard deviation of $4.5 \pm 1 \cdot 10^{8}$ pN; second distribution centered at 16 ± 1 pN with an amplitude of 10.6 ± 3.7 counts and a standard deviation of 4.1 ± 0.8 pN; third distribution centered at 24.0 ± 1.5 pN with an amplitude of 0.8 ± 1.5 counts and a standard deviation of 8.6 ± 16.3 pN . Additionaly, one Gaussian function is fitted with unsatisfactory results as well (Figure S7B): .

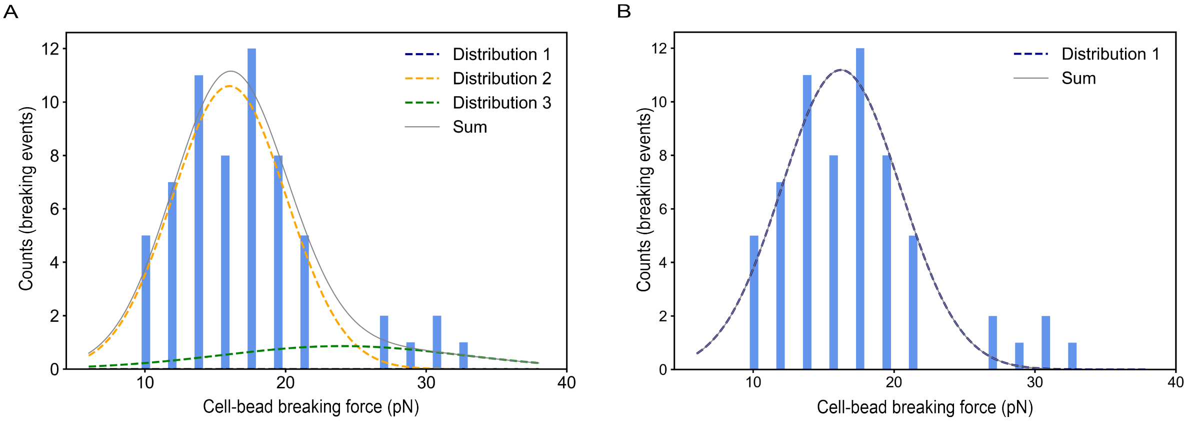

Figure S7. Alternative models used for the bond breaking force distribution
(A) Fitting the data to a sum of three Gaussian functions (note that distribution 1 has such small amplitude that it is barely visible).
(B) Fitting the data to a single Gaussian function.

Determining the binding kinetics

Binding kinetics can be determined from the bond breaking force similar as in[27]. First, determining k_{off} is attempted by plotting the mean adhesion force (F_b) as a function of the logarithm of the loading rate (r):

$$k_{off} = (r_{(F=0)} \cdot x_\beta)/(k_B \cdot T) \tag{S26}$$

From the fit (Figure S8A), the slope is not significantly different than 0, and due to instrument resolution, ultimately the best estimate is that k_{off} should be assumed zero, i.e., adhesins do not spontaneously disengage.

Then, to determine k_{on}, the probability of binding for various contact times between the bead and the cell is calculated:

$$k_{on} = t_{0.5}{}^{-1} \cdot N_A \cdot V_{eff} \tag{S27}$$

The graph (Figure S8B) then did not allow a meaningful measurement of k_{on} (compared to[27], which is due the used instrument time resolution of 1 s while typical on-rates are expected to be in the order of 100 ms). Therefore only the

corresponding bounds can be estimated, i.e., $t_{0.5} < 1$ and therefore $k_{on} > 1.5\ M^{-1}s^{-1}$.

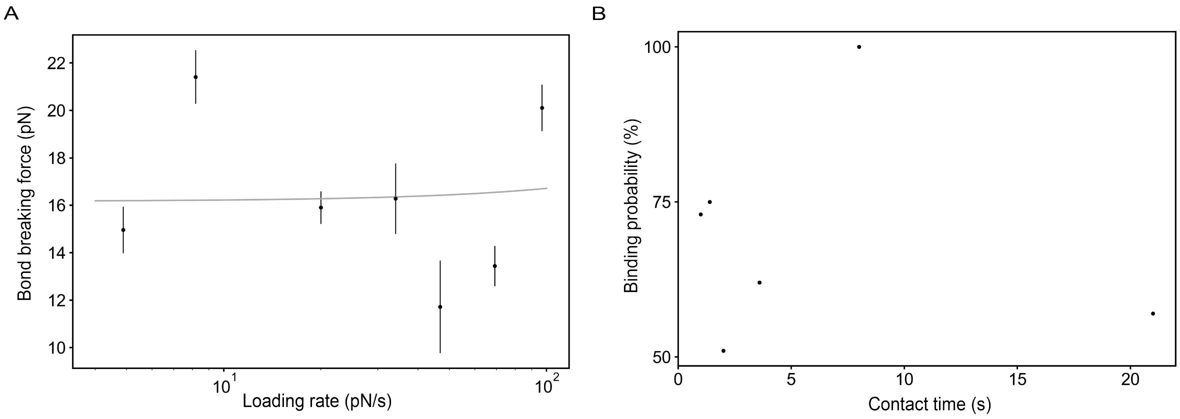

Figure S8. The binding kinetics can be determined from the bond-breaking force
(A) Fitting the the mean adhesion force as a function of the logarithm of the loading rate (r).
(B) Plotting the probability of binding (equivalent to the bond-breaking events) as a function of contact time.

Error Analysis:

Random error:

Through repeated measurements, the random error was determined to be:

$$F_b = 16.2 \pm 0.4 \text{ pN}$$

Systematic error:

- Errors associated with image analysis: The camera's pixel area is $A_{pix} = 66.6^2\ nm^2$, which causes slight variations in the image masking and which then causes the bead center of illumination to vary in the x-y plane. The data has a standard deviation of ±8.8 nm in bead center location (calculated from 1,810 points of data). All analysis occurs from the image processing of individual frames in which a breakage event occurs. When a breakage event occurs, it is unclear whether the breakage event happened at the beginning of the frame or the end. Therefore, the mid point is assumed in all of the data shown above. Due to this, there is an error which scales based on the frame rate, F_r (in frames per second) and the loading rate (F_{rate}). The maximum amount of error is centered around the reported values and is calculated as: $Error_{max-OT} = F_{rate}/2F_r$. F_r was kept at 10/s, and the load rate was varied between 5 and 100 pN/s. For a typical value, this leads to maximum error of $Error_{max-OT} \sim \pm 1$ pN.
- There is 5 % transverse trapping efficiency variability due to the variability in bead size, which would translate to 5 % systematic error in the measured bond breaking force.

  Together these errors add up to an estimated systematic error of:

$$F_b = 16.2 \pm 1.8 \text{ pN}$$

Supplemental Note 5: Predicting and tuning ELM properties

Determining the length of the molecular bond between two cells due to a synthetic adhesin pair

The nanobody length is 125 amino acids (aa) which roughly translates to 13 kDa and it has a length of 4 nm[28]. The length of the p-53 antigen is roughly the same. Therefore the total bond length was estimated to be 2M = 8nm (see Figure S9 for illustration).

Excess tensile strength in dependence of induction level

The cell culture is centrifuged in order to get a high-density cell pellet which is then extruded through a syringe with a needle with inner diameter of 0.8 mm and at a constant flow rate. The material string ruptures at a certain length which is directly related to the tensile strength.

In Figure 6C (main paper), the following tensile strengths $\sigma(C)$ in dependence of induction level C were measured (always mean $\pm$ std):

$\sigma(0) = 1.37 \pm 0.09$ kPa

$\sigma(100) = 2.61 \pm 0.14$ kPa

$\sigma(300) = 3.53 \pm 0.16$ kPa

Here, $\sigma(0)$ is not zero as there are molecules other than the synthetic adhesins than can mediate binding between cells. For non-zero induction level, the difference to zero induction level is then termed ”excess tensile strength”, i.e., $\delta(C) = \sigma(C) - \sigma(0)$.

Based on Eq. 1 main paper, we then test whether $\delta(C)$ is proportional to the corresponding synthetic adhesin level, i.e., whether

$$\delta(C) \sim N_{adh}(C) \tag{S28}$$

With (see Eq. 1)

$$N_{adh}(C) \sim \frac{C^n}{C^n + C_{1/2}^n} \tag{S29}$$

we find that

$$\frac{\sigma(C_1) - \sigma(0)}{\sigma(C_2) - \sigma(0)} = \frac{\delta(C_1)}{\delta(C_2)} = \frac{C_1^n \cdot (C_2^n + C_{1/2}^n)}{C_2^n \cdot (C_1^n + C_{1/2}^n)} \tag{S30}$$

and where C_1 = 100 ng/ml and C_2 = 300 ng/ml refer to the two different inducer concentrations that were tested experimentally.

Using the values as determined in the paper (i.e., $C_{1/2} = 85.6 \pm 6.5$ ng/mL, and $n = 1.8 \pm 0.1$), we then find for the left and right side of Eq. S30 values of 0.57 ± 0.07 and 0.63 ± 0.03, respectively, which according to a t-test can be considered not to be statistically different (p = 0.2).

In conclusion, under the current experimental conditions, the measured ”excess tensile strength” appears to be proportional to the number of expressed synthetic adhesin.

Determining the ordering of cell packing inside the material string

A cell strain expressing homophilic adhesins is transformed with a fluorescent plasmid. The resulting material string is then placed in a slide chamber with a height equal to the needle diameter and imaged using confocal microscopy. The images are taken at different depths of the material string. The orientation of the cells relative to the string orientation in the images is measured using the angle tool in ImageJ. The values of the measured angles from 30 cells are then used to calculate the circular mean, $Z_{exp} = 0.05$ rad. Note that only angles within the 0-180 degrees interval can be measured, as cells are symmetric under 180 degree rotation. In order to be able to compute a meaningful circular mean, 180 degrees are randomly added to half of the data. Bootstrapping is then used to determine the uncertainty in the results, leading to a random error of 0.03 rad. This experimental circular variance is then compared to a synthetic data set, where 30 cells were randomly assigned an angle between 0 and 360 degrees. Then the same analysis is performed as for the experimental data, leading to $Z_{syn} = 0.06 \pm 0.03$ rad (error determined by running the simulation 10 times). A Watson-Williams statistic test is then performed on both data sets, leading to a p value of 0.8, indicating that the circular variance is not

significantly different in both cases. As illustrative counter example, a synthetic data set of 30 angles between 60 and 120 degrees was generated, leading to a circular mean $Z_{syn} = 0.96$, which is significantly higher than the experimental one. Hence no significant alignment order between cells is detected, and it can be considered reasonable to use a model for randomly oriented (disordered) spherocylinders[29] for the following analysis.

Estimating the effective contact area between cell pairs

In the following, several cases are considered where two cells are in contact with each other but have different relative orientation (Figure S9). The corresponding absolute contact area A_{con} (in units of μm^2) is then different in these various cases, and where A_{con} is determined by the length of the Ag-Nb bond (2M) as well as the cellular geometry and cellular dimensions overall. We then define the fractional contact area (in units of 1) with respect to the total surface area of the rod shaped cell ($A_{cell} = 2\pi rl$):

$$\mathscr{A}_{con} = \frac{A_{con}}{A_{cell}} \tag{S31}$$

For the following calculations we use the values of 2M = 8 nm, L = 1 μm, l = 1.8 μm and r = 0.4 μm (see above and Table S3).

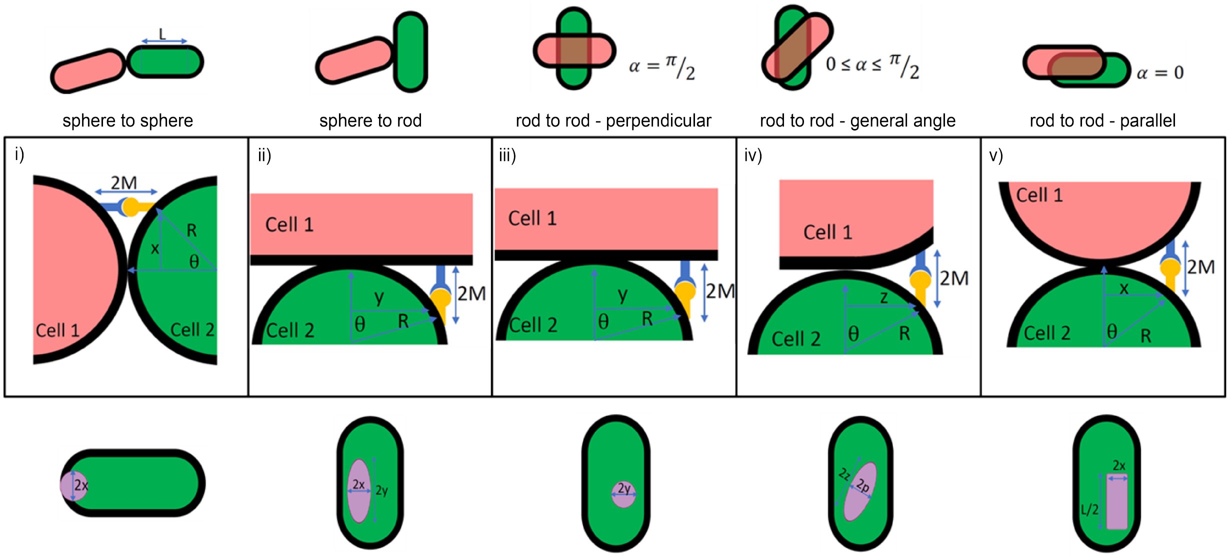

Figure S9. Examples of cell orientations in disordered cell packing

(Top row) Five different orientations for cell to cell connections.

(Middle row) The associated approximate surface area of connection due to bond length (2M), cell radius (r), cylindrical portion of cell length (L), total cell length l=2r+L, the angle between long axes of two cells (α) (for cases iii and iv), and the relative overlap between the two parallel rods (case v).

(Bottom row) The surface area of connection between cells (depicted as purple areas); for cases ii) - iv), the surface areas was estimated as 2D projections of ellipse which defines the potential area of connection between the two cells.

Case i represent two rod shaped cells touching end to end ('ee'):

$$A_{ee} = 2\pi rM = 0.010\ \mu m^2 \tag{S32}$$

$$\mathscr{A}_{ee} = 2\pi rM/(2\pi rl) = M/l = 0.002 \tag{S33}$$

Case ii represents a sphere to rod connection, where a cell's spherical end is touching the cylindrical side of the second cell ('es'):

$$A_{es} = 2\pi M\sqrt{2r(r-M)} = 0.014\ \mu m^2 \quad (S34)$$

$$\mathscr{A}_{es} = 0.003 \quad (S35)$$

Case iii represents a connection between two perpendicular rods ('per') at an angle of $\alpha = \pi/2$ between their long axes (this assumed shape is a rhombus instead of the actual shape and the surface is not wrapped over a cell cylinder, instead a 2D projection is used):

$$A_{per} = 4\pi M(r-M) = 0.021\ \mu m^2 \quad (S36)$$

$$\mathscr{A}_{per} = 0.005 \quad (S37)$$

Case iv represents a connection between two cells oriented at some angle α ('ang') where between their long axes will be an ellipse with width 2p and length 2z (this assumed shape is a rhombus instead of the actual shape, and the surface is not wrapped over cell cylinder, instead a 2D projection is used):

$$A_{ang} = \pi[\sqrt{2rM-M^2}\cos(\alpha) + (\sqrt{4rM-M^2}\sin(\alpha)] \times [\sqrt{4rM-M^2}\sin(\alpha) + (\frac{L}{2})\cos(\alpha)] = 0.056\ \mu m^2 \quad (S38)$$

$$\mathscr{A}_{ang} = 0.013 \quad (S39)$$

In order to estimate the expected value of surface area between this type of connection, the average over 0 to $\pi/2$ was used, i.e., $\alpha = \pi/4$.

Case v represents two parallel rods ('par') that are assumed to be attached to each other over ~100% of the length of cylindrical part of the cell ($L = l - 2r$):

$$A_{par} = rL(\cos^{-1}(\frac{r-2M}{r})) = 0.165\ \mu m^2 \quad (S40)$$

$$\mathscr{A}_{par} = 0.036 \quad (S41)$$

If the rods' overlap is less, then area decreases by the corresponding fractional amount, e.g., if overlap is only 50 %, then A_{par} = 0.083 μm^2 and $\mathscr{A}_{par} = 0.018$.

The values for $\mathscr{A}_{con}$ for all cases are then also listed in Figure 6F (main paper).

Average all cases: To obtain a first-pass estimate of the average contact area between cells in a disordered system, one can average over all these contact areas (and where for case ii we assume 50 % overlap as the more disordered scenario):

$$A_{avg} \approx 0.04\ \mu m^2 \quad (S42)$$

$$\mathscr{A}_{avg} \approx 0.008 \quad (S43)$$

Model for predicting the excess tensile strength of the engineered living material string

This excess tensile strength for any induction concentration C is then given by

$$\delta_{model}(C) = N_{adh,br}(C) \cdot F_b \cdot N_{cell}/A_s \quad (S44)$$

where N_{cell} is the number of cell pairs that are broken apart in the rupture event, $N_{adh,br}(C)$ is the number of synthetic

adhesins that connect both cells before breaking and which depends on induction level, F_b is the bond breaking force between any adhesin pair, and A_s is the cross sectional area of the material string.

$N_{adh,br}(C)$ then depends on the total number of adhesins per cell, $N_{adh}(C)$, multiplied with the effective fractional area $\mathscr{A}_{con}$ of the contact point between any two cells that gets broken (which generally depends on cell alignment direction for ordered materials relative to direction of applied force, or it is some average over many connection types for disordered materials), and the number of such contact points that a cell has with other cells and that actually get broken (which again depends on the amount and type of ordering in the material; in the following we assume for simplicity only the ordered case of a 'primitive tetragonal lattice'[30], and hence the number of contact points to be broken between any two cells is one).

The material consists of homophilic cells, where the Nb is encoded on a medium copy plasmid (20-30 copy number[31]) and the Ag is encoded on a low copy plasmid (10-12 copy number[31]) (Methods). Therefore the number of expressed Ag is the limiting factor in the number of connections between any two cells, and a correction factor $f_p = 0.5$ is needed to correct the number of adhesins $N_{adh}(C)$ as we previously determined using a medium copy plasmid.

Hence we find:

$$N_{adh,br}(C) = f_p \cdot N_{adh}(C) \cdot \mathscr{A}_{con} \tag{S45}$$

which leads to:

$$\delta_{model}(C) = f_p \cdot N_{adh}(C) \cdot \mathscr{A}_{con} \cdot F_b \cdot N_{cell}/A_s \tag{S46}$$

In general, N_{cell} is proportional to A_s, i.e., the tensile strength is expected to not depend explicitly on A_s.

The ratio N_{cell}/A_s can be estimated as follows: Consider a primitive tetragonal lattice with two mono-layers of cells on top of each other and that are perpendicular to string with an area of A_s and height h (Figure S10). These two monolayers then contain the N_{cell} pairs, i.e., each monolayer contains N_{cell} cells. During the string breakage event, these two monolayers are separated. The total volume of one monolayer then is:

$$V_{layer} = A_s \cdot h = N_{cell} \cdot V_{cell}/f_{sv} \tag{S47}$$

with V_{cell} being the volume of an individual cell, and f_{sv} the solid to volume fraction of the material (a unitless number between 0 and 1), i.e., each cell actually requires a volume of V_{cell}/f_{sv} to fit inside the material.

f_{sv} for either vertically or horizontally aligned cells is approximately equal to the packing efficiency of a crystalline phase in a system of parallel hard spherocylinders, 0.8[32], whereas for disordered packing of cells with aspect ratio of l/(2r) is 0.6[29]. The remaining fraction is occupied by the pores between cells which can be either filled by air or medium (water). From f_{sv} and the aspect ratio we can also estimate the number of neighbors a cell can come into contact with, $N_c = 4$[33]:

$$N_c = f_{sv} \cdot \frac{l}{2r} \cdot \left(4\frac{2r}{l} + \frac{3 \cdot l/2r}{3 \cdot l/2r + 2}\right) \tag{S48}$$

The relationship in Eq. S47) can the be converted to:

$$N_{cell}/A_s = f_{sv} \cdot h/V_{cell} \tag{S49}$$

The height h itself depends on how cells are arranged inside the material. Note also that effectively $2r \le h \le l$, and for the different cases h becomes:

Case A: Cells are perfectly stacked in a primitive tetragonal lattice, and their their long axis is aligned perpendicular to the rupture plane (Figure S10A).

$$h_{per} = l \tag{S50}$$

Case B: Cells are perfectly stacked in a primitive tetragonal lattice, and their long axis is aligned in parallel with the rupture plane (Figure S10B).

$$h_{par} = 2r \tag{S51}$$

Case C: Cells are randomly arranged (disordered), in which case we assume for simplicity the average of two ordered cases:

$$h_{avg} = \frac{(h_{par} + h_{per})}{2} = \frac{2r + l}{2} \tag{S52}$$

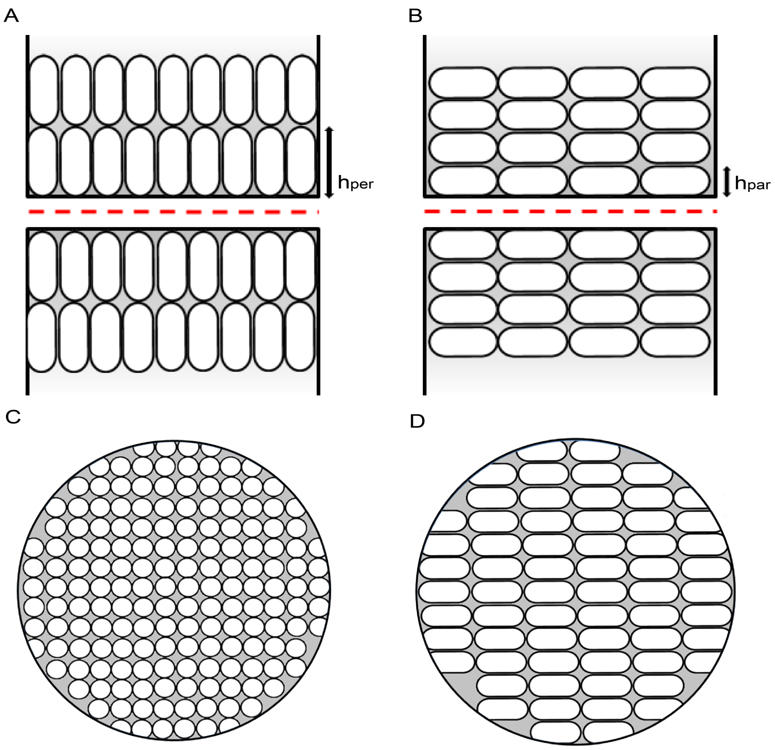

Figure S10. Schematic of the material string rupturing (rupture plane in red line; assuming a primitive tetragonal lattice)
(A) Long axis of cells aligned perpendicular with the rupture plane; the height of the monolayer is $h_{per} = l$ µm.
(B) Long axis of cells aligned in parallel with the rupture plane; the height of the monolayer is $h_{par} = 2r$ µm.
(C) The top view of the cross-sectional area of the string where the long axis of cells is aligned perpendicular with the rupture plane (corresponds to the lateral view in (A)).
(D) The top view of the cross-sectional area of the string where the long axis of cells is aligned in parallel with the rupture plane (corresponds to the lateral view in (B)). (Boundary effects can be neglected, i.e., on the relevant length scales the cells are not cut off at the string circumference as seen here, and the circumference can be considered smooth.) Cells and material string not drawn to scale.

Finally, we insert the newfound relation for N_{cell}/A_{str} in Eq. S46 to find the following relation for the excess tensile strength:

$$\delta_{model}(C) \quad = \quad f_p \cdot N_{adh}(C) \cdot \mathscr{A}_{con} \cdot F_b \cdot f_{sv} \cdot h/V_{cell} \tag{S53}$$

Hence the final result also highlights the four key contributions to excess tensile strength, i.e., the number of adhesin pairs per cell ($f_p \cdot N_{adh}(C)$), which is correspondingly reduced due to cell ordering and packing inside the material ($f_{sv} \cdot \mathscr{A}_{con}$), the bond breaking force per adhesin pair (F_b), and the physical dimensions of the cell (h/V_{cell}, which represents the effective area a cell takes up in the rupture plane, thereby determining how many cells can be packed into this plane,

Predicting the excess tensile strength of the material based on the model for an induction level of 100 ng/mL aTc

For the next calculation we use the following parameters that had all been determined previously:

$$
\begin{aligned}
f_p &= 0.5 \\
N_{adh}(100) &= 7,300 \pm 1,800 \text{ adhesins/cell} \\
F_b &= 16.2 \pm 0.4 \text{ pN} \\
l &= 1.80 \pm 0.25\ \mu\text{m} \\
r &= 0.4 \pm 0.1\ \mu\text{m} \\
V_{cell} &= 0.96 \pm 0.14\ \mu\text{m}^3
\end{aligned}
$$

The theoretical expectation of the excess tensile strength of the material string due to the synthetic adhesins (for a syringe needle with a diameter of 0.8 mm and an induction level of 100 ng/mL aTc) is then determined through a Monte-Carlo simulation (mean and std) for the following cases:

CASE A: The material consists of cells that are perfectly stacked and aligned perpendicular to the rupture plane (Figure S10A), hence $\mathscr{A}_{con} = \mathscr{A}_{ee} = 0.002$, $h = h_{per} = l$, and $f_{sv} \approx 0.8$[32]:

$$\delta_{per}(100) = 0.17 \pm 0.03 \text{ kPa} \tag{S54}$$

CASE B: The material consists of cells that are perfectly stacked and aligned in parallel to the string (Figure S10zb), hence $\mathscr{A}_{con} = \mathscr{A}_{par} = 0.036$ (where the overlap between cells is maximal, i.e., 100%), $h = h_{par} = 2r$, and $f_{sv} \approx 0.8$[32]:

$$\delta_{par}(100) = 1.70 \pm 0.25 \text{ kPa} \tag{S55}$$

CASE C: The material consists of cells that are disordered (Figure 6E), hence $\mathscr{A}_{con} = \mathscr{A}_{avg} = 0.008$, $h = h_{avg} = (2r+l)/2$, and $f_{sv} \approx 0.6$[29]:

$$\delta_{avg}(100) = 0.50 \pm 0.07 \text{ kPa} \tag{S56}$$

For comparison, the experimental results reveal that the difference in tensile strength between the induced and uninduced cells is:

$$\delta_{exp}(100) = 1.2 \pm 0.2 \text{ kPa} \tag{S57}$$

Hence the experimental results is situated between the two extreme cases ($\delta_{per}(100)$ and $\delta_{par}(100)$, which span an order of magnitude) and is approximately within a factor of 2 to the predicted excess tensile strength for disordered packing ($\delta_{avg}(100)$) (Figure 6G), which is considered a satisfactory agreement for such order of magnitude estimates. Note also that ordered (crystalline) packings[30] can have even higher densities overall including multiple contact points to be broken per cell, hence the theoretical values could then be a factor of about 2-3 higher - the corresponding mathematical analysis for the various cases is left to the interested reader.

Predicting the excess tensile strength of the material based on the model for an induction level of 300 ng/mL aTc

Such agreement also holds true for 300 ng/mL aTc induction when performing the corresponding analysis (Figure S11). This agreement is expected given the earlier analysis result that that the "excess tensile strength" is proportional to the number of synthetic adhesins per cell.

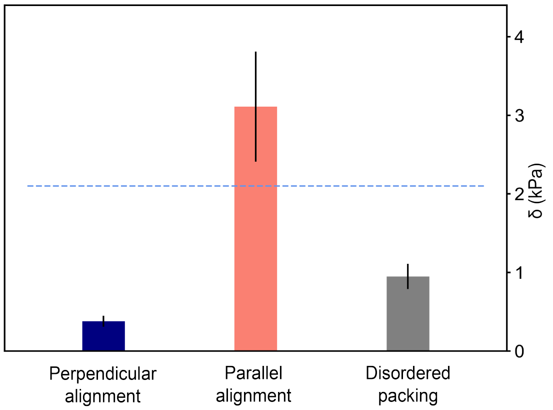

Figure S11. Effective material-level adhesion (comparison modeling and experiment at 300 ng/mL) Different configurations between cells inside the material due to the assumed packing and ordering: perfect perpendicular alignment to the rupture plane model (dark blue) vs. parallel alignment to the rupture plane model (orange) as extreme cases; a more realistic model (grey) based on averaging over many possible configurations; experimental results from Figure 6C (blue horizontal line).

Additional control experiments

1. **The evaporation of water** does not meaningfully affect our tensile strength measurements, as for the duration of a rupture event of ~ 1 minute, the mass loss due to evaporation is less than 5 % (Figure S12). This was measured by laying the material string on a string, then doubling the mass loss due to evaporation by 2 to account for the actual experimental conditions were the string is suspended in the air.

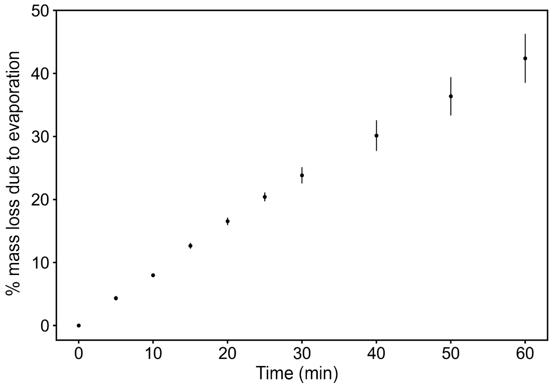

Figure S12. The mass loss of a material string over time increased due to evaporation

2. **The use of a mix of heterophilic adhesin cells**, EPEA and anti-EPEA, even uninduced, leads to a lower rupture force than WT (Figure S13A) (in contrast to the materials investigate so far consisting of a single homophilic cell type that expresses both Nb and Ag). This suggests that mixtures of different cell types leads to inhomogeneities in the material, lowering the overall material integrity (such as tensile strength). In agreement with that notion, when comparing pictures of the string (Figure S13B), there are inhomogeneities present in these mixtures. Hence the emergent properties of composite bacterial material are left for future investigations.

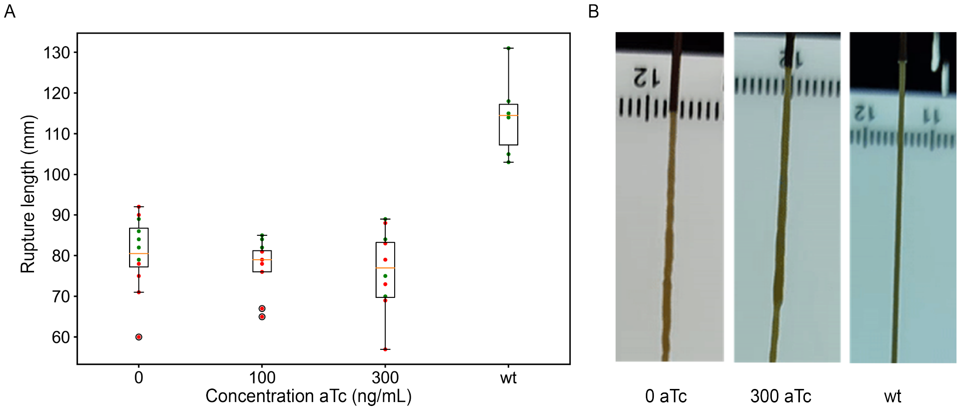

Figure S13. Investigating the use of a heteroophilic cell mixture in ELM preparation
(A) Comparison in the rupture length between the wt cell string and the string consisting of heterophilic antiEPEA/EPEA cells.
(B) Pictures of the ejected string showing inhomogeneities in the material.

Supplemental Note 6: ELM stability and self-regenerability

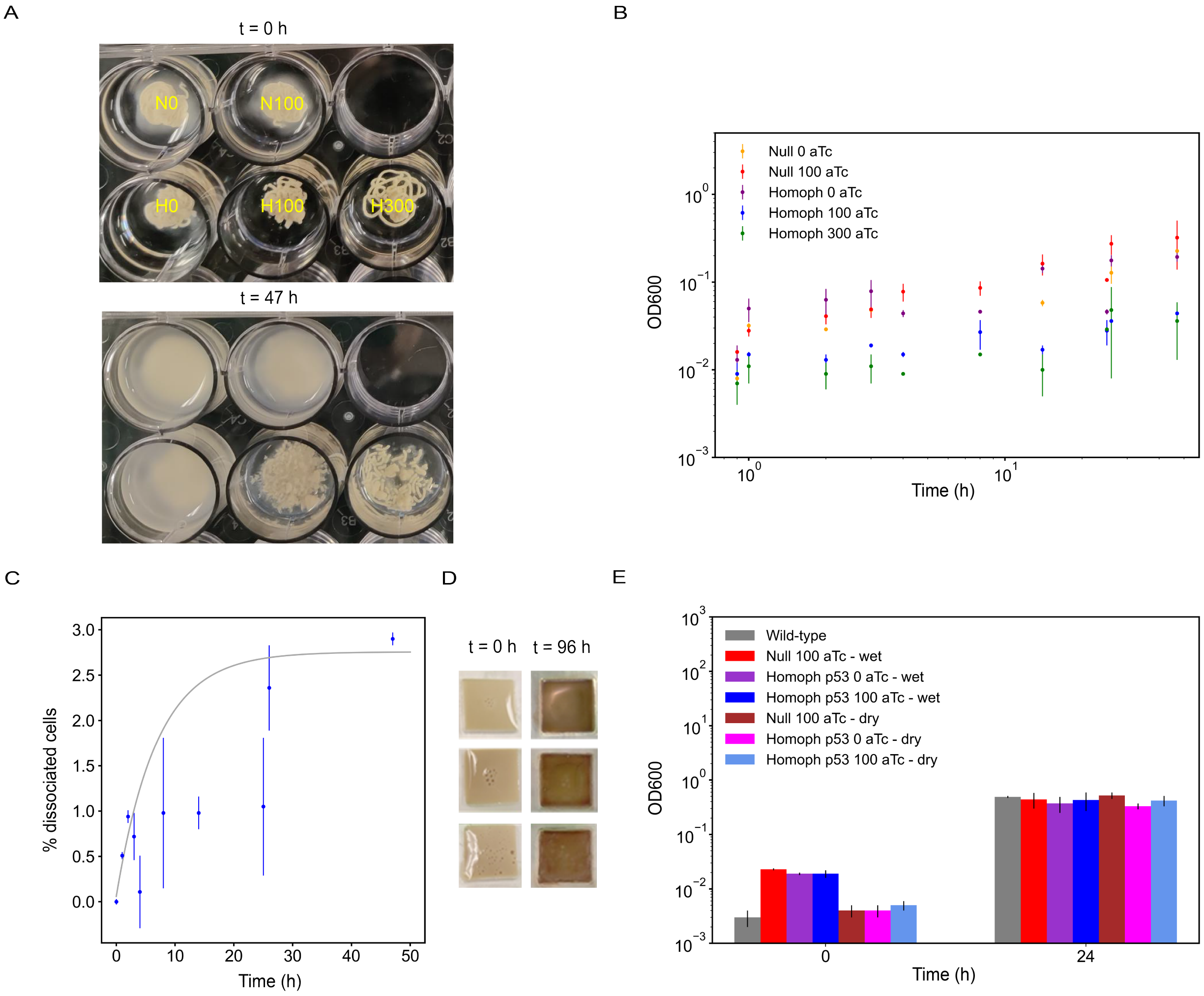

Figure S14. Stability and self-regenerability properties of the adhesin ELM
(A) The ELM string consisting of different cell constructs (N and H standing for the null (control cells containing the autotransporter but no adhesin) and the homophilic p53 adhesin cells respectively, and 0, 100, 300 representing the induction level in ng/mL aTc) was ejected into PBS and shaken at a constant speed for 47 h.
(B) The stability of the ELM measured through the optical density at 600 nm of the solution over time (samples taken from (A) at corresponding time points).
(C) The percentage of homophilic p53 cells induced with 100 ng/mL aTc (blue data poins in (B)) that dissociate from the material string fitted with an exponential equation (grey line).
(D) The ELM cell pellet placed into molds and left to dry for 96 h.
(E) The viability of the cells and the self-regenerability property of the material determined through the OD600 of the cell cultures. Measurements 24 h after inoculation with either the material string left in PBS for 96 h (as in (A)) or with the dried out material for 96 h (as in C) showed significant regrowth for all samples.

First, we investigated the stability of the ELM over time (Figure S14A,B). We prepared materials with different cell constructs and induction levels (with or without adhesins), and we injected these samples in volumes of 0.5 mL in the form of a material string into a dish containing 3 mL PBS (Figure S14A). There were no nutrients or inducer in the solution and consequently the cells did not divide, nor were additional adhesins produced. As cells dissociated from the material over time, the PBS solution became cloudy, which was then quantified from the optical density of the solution.

We monitored the optical density at 600 nm of the (upper part of the well) PBS solution over time, while the wells were shaken at a constant speed of 60 rpm (Figure S14B). To minimize the systematic errors associated with concentrating the solution by taking out the samples at various time points, we gently added back the sample after each measurement without disturbing the cell string. We found a significant difference between null/uninduced cells and the induced cells (Student's t-test, $p \leq 0.05$), such as, at higher inductions, the cell do not break away from the string as much as the lower induction and therefore, the OD600 of the solution is lower (Figure S14B) ($n = 4$ samples). The material string consisting of cells that were expressing adhesins broke along the length but retained its shape after 47 h. We therefore conclude that the ELM made of adhesins expressing cells is stable for several days compared to non-adhesive cells.

We were then interested whether the time scale over which the material degrades can be related to the degradation rate of the synthetic adhesins. Therefore, we calculated the percentage of cells that dissociated from the string as $(T_x - T_0)/T_f \cdot 100\%$, where $T_0, T_x,$ andT_f represent the OD600 of the PBS solution at time 0, x hours and at the end when the string was mixed with PBS into a homogeneous solution, respectively (Figure S14C). We then fitted this data to an exponential equation, leading to a falling off rate of 0.14 ± 0.05 h^{-1}. This is within a factor of three compared to the degradation rate of the adhesins of 0.05 ± 0.01 h^{-1} that was previously determined. Hence the material presents similar degradation scales as the synthetic adhesins.

Next, we investigated the longevity and the capacity to self-regenerate of the adhesin-expressing cells (Figure S14D,E). We prepared material pellets with different constructs and induction levels as before and performed two experiments in parallel: (i) The cell pellet was placed in molds on a special membranes, dried out for 96 h[34] (Figure S14D) and then rehydrated and diluted for cell growth; (ii) a string of material was placed into PBS for 96 h and then a sample was taken out and placed in LB for growth. In both cases, the cells grew back to a final OD600 similar to wild type cells grown under normal conditions (Student's t-test, $p > 0.2$) (Figure S14E) ($n = 2$ samples each setup). We conclude that the cells comprising the ELM have a longevity for at least multiple days under these conditions, and that the material is capable of self-regeneration due to capability of cell division and cell growth.

Supplemental references

1. Gregor, T., Tank, D. W., Wieschaus, E. F., and Bialek, W. (2007). Probing the limits to positional information. Cell *130*, 153–164. doi:10.1016/j.cell.2007.05.025.

2. Cole, R. W., Jinadasa, T., and Brown, C. M. (2011). Measuring and interpreting point spread functions to determine confocal microscope resolution and ensure quality control. Nature protocols *6*, 1929–1941. doi:10.1038/nprot.2011.407.

3. Hughes, I., and Hase, T. (2010). Measurements and their uncertainties: a practical guide to modern error analysis.

4. Mechels, S. E., and Young, M. (1992). Scanning confocal microscope for accurate dimensional measurement. *1660*, 542–550. doi:10.1117/12.59541.

5. Alon, U. (2019). An introduction to systems biology: design principles of biological circuits.

6. Koch, A. L., and Levy, H. R. (1955). Protein turnover in growing cultures of escherichia coli. Journal of Biological Chemistry *217*, 947–958. doi:10.1016/S0021-9258(18)65958-7.

7. Hogness, D. S., Cohn, M., and Monod, J. (1955). Studies on the induced synthesis of β-galactosidase in escherichia coli: the kinetics and mechanism of sulfur incorporation. Biochimica et biophysica acta *16*, 99–116. doi:10.1016/0006-3002(55)90188-8.

8. Mandelstam, J. (1958). Turnover of protein in growing and non-growing populations of escherichia coli. Biochemical Journal *69*, 110. doi:10.1042/bj0690110.

9. Axelrod, D., Koppel, D., Schlessinger, J., Elson, E., and Webb, W. W. (1976). Mobility measurement by analysis of fluorescence photobleaching recovery kinetics. Biophysical journal *16*, 1055–1069. doi:10.1016/S0006-3495(76)85755-4.

10. Halavatyi, A., and Terjung, S. (2017). Frap and other photoperturbation techniques. Standard and Super-Resolution Bioimaging Data Analysis: A Primer (99). doi:10.1002/9781119096948.ch5.

11. Pincet, F., Adrien, V., Yang, R., Delacotte, J., Rothman, J. E., Urbach, W., and Tareste, D. (2016). Frap to characterize molecular diffusion and interaction in various membrane environments. PloS one *11*, e0158457. doi:10.1371/journal.pone.0158457.

12. Day, C. A., Kraft, L. J., Kang, M., and Kenworthy, A. K. (2012). Analysis of protein and lipid dynamics using confocal fluorescence recovery after photobleaching (frap). Current protocols in cytometry *62*, 2–19. doi:10.1002/0471142956.cy0219s62.

13. Elowitz, M. B., Surette, M. G., Wolf, P.-E., Stock, J. B., and Leibler, S. (1999). Protein mobility in the cytoplasm ofescherichia coli. Journal of bacteriology *181*, 197–203. doi:10.1128/jb.181.1.197-203.1999.

14. Van der Ploeg, R., Verheul, J., Vischer, N. O., Alexeeva, S., Hoogendoorn, E., Postma, M., Banzhaf, M., Vollmer, W., and Den Blaauwen, T. (2013). Colocalization and interaction between elongasome and divisome during a preparative cell division phase in e scherichia coli. Molecular microbiology *87*, 1074–1087. doi:10.1111/mmi.12150.

15. Phair, R. D., Scaffidi, P., Elbi, C., Vecerová, J., Dey, A., Ozato, K., Brown, D. T., Hager, G., Bustin, M., and Misteli, T. (2004). Global nature of dynamic protein-chromatin interactions in vivo: three-dimensional genome scanning and dynamic interaction networks of chromatin proteins. Molecular and cellular biology *24*, 6393–6402. doi:10.1128/MCB.24.14.6393-6402.2004.

16. Bianchi, F., Syga, Ł., Moiset, G., Spakman, D., Schavemaker, P. E., Punter, C. M., Seinen, A.-B., van Oijen, A. M., Robinson, A., and Poolman, B. (2018). Steric exclusion and protein conformation determine the localization of plasma membrane transporters. Nature communications *9*, 501. doi:10.1038/s41467-018-02864-2.

17. Niemela, P. S., Miettinen, M. S., Monticelli, L., Hammaren, H., Bjelkmar, P., Murtola, T., Lindahl, E., and Vattulainen, I. (2010). Membrane proteins diffuse as dynamic complexes with lipids. Journal of the American Chemical Society *132*, 7574–7575. doi:10.1021/ja101481b.

18. Bläßle, A., Soh, G., Braun, T., Mörsdorf, D., Preiß, H., Jordan, B. M., and Müller, P. (2018). Quantitative diffusion measurements using the open-source software pyfrap. Nature communications *9*, 1–14. doi:10.1038/s41467-018-03975-6.

19. Grandbois, M., Beyer, M., Rief, M., Clausen-Schaumann, H., and Gaub, H. E. (1999). How strong is a covalent bond? Science *283*, 1727–1730. doi:10.1126/science.283.5408.172.

20. Pincet, F., and Husson, J. (2005). The solution to the streptavidin-biotin paradox: the influence of history on the strength of single molecular bonds. Biophysical journal *89*, 4374–4381. doi:10.1529/biophysj.105.067769.

21. Lower, B. H., Yongsunthon, R., Vellano III, F. P., and Lower, S. K. (2005). Simultaneous force and fluorescence measurements of a protein that forms a bond between a living bacterium and a solid surface. Journal of Bacteriology *187*, 2127–2137. doi:10.1128/jb.187.6.2127-2137.2005.

22. Klamecka, K., Severin, P. M., Milles, L. F., Gaub, H. E., and Leonhardt, H. (2015). Energy profile of nanobody–gfp complex under force. Physical biology *12*, 056009. doi:10.1088/1478-3975/12/5/056009.

23. Wang, W., Yuan, J., and Jiang, C. (2021). Applications of nanobodies in plant science and biotechnology. Plant Molecular Biology *105*, 43–53. doi:10.1007/s11103-020-01082-z.

24. Koch, M., and Shaevitz, J. (2017). Introduction to optical tweezers. Methods in molecular biology *1486*, 3–24. doi:10.1007/978-1-4939-6421-5_1.

25. Markowska, A., Markowski, A. R., and Jarocka-Karpowicz, I. (2021). The importance of 6-aminohexanoic acid as a hydrophobic, flexible structural element. International Journal of Molecular Sciences *22*, 12122. doi:10.3390/ijms222212122.

26. Neish, C. S., Martin, I. L., Henderson, R. M., and Edwardson, J. M. (2002). Direct visualization of ligand-protein interactions using atomic force microscopy. British journal of pharmacology *135*, 1943–1950. doi:10.1038/sj.bjp.0704660.

27. El-Kirat-Chatel, S., Mil-Homens, D., Beaussart, A., Fialho, A. M., and Dufrêne, Y. F. (2013). Single-molecule atomic force microscopy unravels the binding mechanism of a b urkholderia cenocepacia trimeric autotransporter adhesin. Molecular microbiology *89*, 649–659. doi:10.1111/mmi.12301.

28. Jovčevska, I., and Muyldermans, S. (2020). The therapeutic potential of nanobodies. BioDrugs *34*, 11–26. doi:10.1007/s40259-019-00392-z.

29. Williams, S. R., and Philipse, A. P. (2003). Random packings of spheres and spherocylinders simulated by mechanical contraction. Physical Review E *67*, 051301. doi:10.1103/PhysRevE.67.051301.

30. Kittel, C., and McEuen, P. (2018). Introduction to solid state physics.

31. Lutz, R., and Bujard, H. (1997). Independent and tight regulation of transcriptional units in escherichia coli via the lacr/o, the tetr/o and arac/i1-i2 regulatory elements. Nucleic acids research *25*, 1203–1210. doi:10.1093/nar/25.6.1203.

32. Veerman, J., and Frenkel, D. (1991). Relative stability of columnar and crystalline phases in a system of parallel hard spherocylinders. Physical Review A *43*, 4334. doi:10.1103/PhysRevA.43.4334.

33. Philipse, A. P. (1996). The random contact equation and its implications for (colloidal) rods in packings, suspensions, and anisotropic powders. Langmuir *12*, 1127–1133. doi:10.1021/la950671o.

34. Manjula-Basavanna, A., Duraj-Thatte, A. M., and Joshi, N. S. (2021). Robust self-regeneratable stiff living materials fabricated from microbial cells. Advanced functional materials *31*, 2010784. doi:10.1002/adfm.202010784.
